# Assessment of the clinical efficacy of the heart spectrum blood pressure monitor for diagnosis of atrial fibrillation: An unblinded clinical trial

**DOI:** 10.1371/journal.pone.0198852

**Published:** 2018-06-14

**Authors:** Wei-Fong Kao, Sen-Kuang Hou, Chun-Yao Huang, Chun-Chieh Chao, Chung-Chih Cheng, Yi-Jung Chen

**Affiliations:** 1 Department of Emergency Medicine, School of Medicine, College of Medicine, Taipei Medical University, Taipei, Taiwan; 2 Department of Emergency Medicine, Taipei Medical University Hospital, Taipei, Taiwan; 3 Division of Cardiology and Cardiovascular Research Center, Department of Internal Medicine, Taipei Medical University Hospital, Taipei, Taiwan; 4 Department of Internal Medicine, School of Medicine, College of Medicine, Taipei Medical University, Taipei, Taiwan; 5 Department of Medical Device, Medical and Pharmaceutical Industry Technology and Development Center, Taipei, Taiwan; Kurume University School of Medicine, JAPAN

## Abstract

Atrial fibrillation (AF) is the most common arrhythmia. The most common diagnostic method, 12-lead electrocardiogram (ECG), can record episodes of arrhythmia from which the type and severity can be determined. The Heart Spectrum Blood Pressure Monitor (P2; OSTAR Meditech Corp., New Taipei City, Taiwan) is used to measure cardiovascular pressure change with fast Fourier transform (FFT) analysis to obtain heart rate frequency variability and accurate blood pressure data. We compared the diagnostic efficacy of the Heart Spectrum Blood Pressure Monitor to a 12-lead ECG (gold standard) for patients with AF. Three measurement methods were used in this study to analyze the heart index and compare the results with simultaneous 12-lead ECG: blood pressure; mean arterial pressure, which was calculated from individual blood pressure as a constant pressure; and a constant pressure of 60 mmHg. The physician used a 12-lead ECG and the Heart Spectrum Blood Pressure Monitor simultaneously. The Heart Spectrum Blood Pressure Monitor used FFT analysis to diagnose AF, and the findings were compared to the 12-lead ECG readings. This unblinded clinical trial was conducted in the emergency department of Taipei Medical University Hospital. Twenty-nine subjects with AF and 33 without AF aged 25 to 97 y (mean, 63.5 y) were included. Subjects who were exposed to high-frequency surgical equipment during testing, those with cardiac pacemakers or implantable defibrillators, and pregnant women were excluded. The sensitivity, specificity, positive predictive value (PPV), and negative predictive value (NPV) were 97%, 97%, 97%, and 97%, respectively, for method 1; 90%, 100%, 100%, and 91%, respectively, for method 2; and 100%, 94%, 94%, and 100%, respectively, for method 3. The sensitivity, specificity, PPV, and NPV for both methods ranged between 90% and 100%, indicating that the Heart Spectrum Blood Pressure Monitor can be effectively applied for AF detection.

## Introduction

### General background information

Atrial fibrillation (AF) is a common cardiac arrhythmia. An estimated 2.2 million people (median age, approximately 75 y) in the United States have AF [1]. It is worth noting that from 1985 to 1999, hospitalizations for a first diagnosis increased from 154,086 to 376,487 [2]. Overall, the age-standardized rate (per 100,000) increased from 27.6 in 1980 to 69.8 in 1998 (an average annual increase of 5.4%; *p* < 0.0001) [3].

In general, the occurrence of AF with atrial degeneration is related; therefore, the older the population, the higher the incidence of AF. The estimated prevalence of AF is 0.4% to 1% in the general population, and it increases with age, up to 5% to 6% for those older than 65 y and up to 8% among those 80 y or older. Similarly, although the incidence of AF is <0.1% annually for those younger than 40 y, it increases to >1.5% annually for women and 2% for men older than 80 y [4].

The incidence of stroke is more than four times higher for patients with cardiac failure (*p* < 0.001), and five times higher for those with AF (*p* < 0.001) [5]. Patients with AF require medical treatment to prevent the occurrence of stroke. AF was associated with an odds ratio (OR) for death of 1.5 (95% confidence interval [CI], 1.2 to 1.8) for men and 1.9 (95% CI, 1.5 to 2.2) for women [6].

With the aging population, the number of people experiencing AF continues to increase. The age-adjusted incidence of stroke has more than doubled for patients with coronary heart disease, and has more than tripled for those with hypertension. For those with coronary heart disease or cardiac failure, AF doubles the risk of stroke for men and triples it for women [5–9].

Evaluation using 12-lead and dynamic electrocardiogram (ECG) can diagnose most cases of AF [10–12]. However, 12-lead ECG needs to be performed and interpreted at a medical institution by physicians; this cannot be implemented by the user in the home environment.

The Heart Spectrum Blood Pressure Monitor (P2; OSTAR Meditech Corp., New Taipei City, Taiwan) is a simple home-use device that can detect the pressure change in the blood vessels of the heart. The measurement undergoes fast Fourier transformation (FFT) to calculate the heartbeat frequency and obtain accurate blood pressure data. It can also analyze the heartbeat frequency to determine if AF has occurred.

### Research purpose

The purpose of this clinical trial was to compare the diagnostic efficacy of the Heart Spectrum Blood Pressure Monitor and 12-lead ECG for verification of AF. Additionally, an assessment of the clinical efficacy of both methods for the diagnosis of AF was performed.

## Materials and methods

The authors confirm that all ongoing and related trials for this intervention are registered.

### Research design

This clinical trial was conducted at Taipei Medical University Hospital (single-center clinical trial). Sixty-three patients were enrolled during the trial period of approximately 1 y. The trial is registered with ClinicalTrials.gov as Identifier NCT03095131 (https://clinicaltrials.gov/ct2/show/NCT03095131). The study design (S1 and S2 Files) was approved by the Taipei Medical University Joint Institutional Review board (approval no. N20151006) and was conducted according to the principles expressed in the Declaration of Helsinki. The clinical trial at the hospital was to be completed within a limited time frame; therefore, we registered the study after subject enrollment began.

This clinical trial was conducted as an open study (unblinded). At the emergency department, when medical staff members recruited patients with AF or without AF, they alerted the physician on duty to examine the case. The Heart Spectrum Blood Pressure Monitor was compared to 12-lead ECG (ELI 250; Mortara Instrument, Milwaukee, WI, USA) after written informed consent was obtained from the patient. Results were interpreted by the examining physician [13–19]. Finally, the sensitivity, specificity, positive predictive value (PPV), and negative predictive value (NPV) were analyzed to evaluate clinical efficacy.

### Subjects and materials

**Fig 1:**
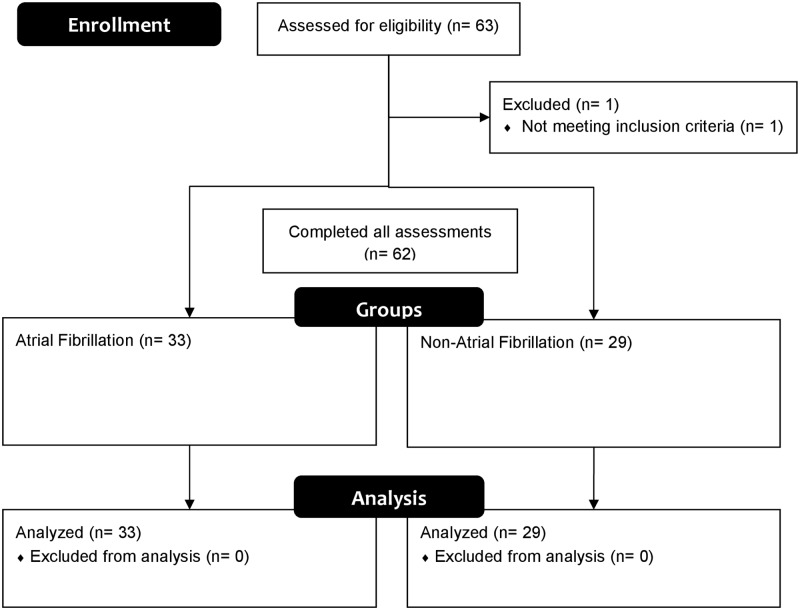
CONSORT flow diagram.

Subjects older than 20 y who were diagnosed with AF using 12-lead ECG or who were healthy (without AF) were included. Each trial was conducted 10 min after diagnosis by a physician. Subjects who had been exposed to high-frequency surgical equipment during testing, those with cardiac pacemakers or implantable defibrillators, and pregnant women were excluded. The complete date range for subject recruitment and follow-up was February 22, 2016 to June 23, 2016. The CONSORT flow diagram for the study is presented in Fig 1.

### Trial and treatment procedures

The physician applied the 12-lead ECG patches to the subject’s body, and attached the blood pressure cuff to the subject’s arm. Then, the Heart Spectrum Blood Pressure Monitor was connected to a computer for data collection. The 12-lead ECG and Heart Spectrum Blood Pressure Monitor were used simultaneously. The Heart Spectrum Blood Pressure Monitor calculated the heart spectrum results via FFT analysis for the diagnosis of AF. This analysis method was invented by OSTAR Meditech Corp. (USA patent number: US 7,107,094 B2; Taiwan invention patent number: I280119). Clinical trial results were judged by the examining physician.

### Methods and analysis

#### Heart spectrum calculation method

**Fig 2:**
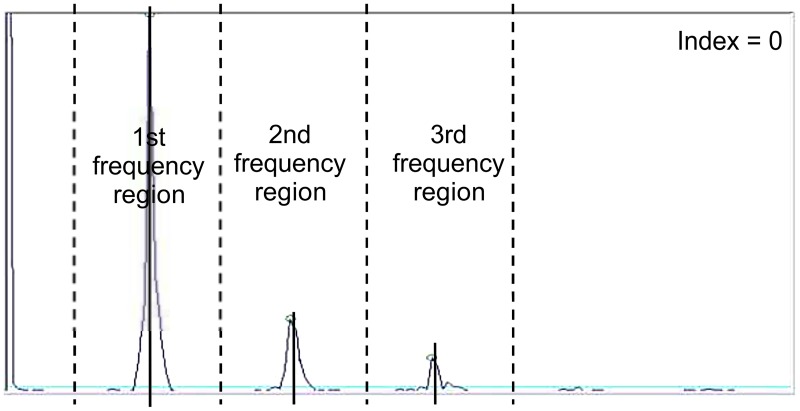
Normal heart spectrum with heart index = 0 (no noise).

**Fig 3:**
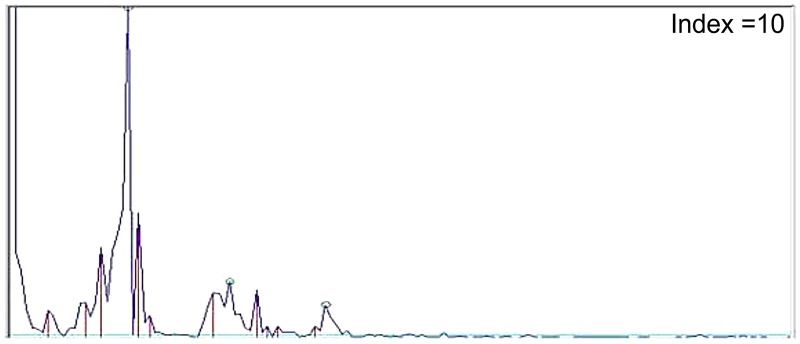
Heart spectrum with heart index = 10.

Human blood pressure and heart rate were measured using the oscillometric method. Each heartbeat causes the heart to emit blood, and then the sensor of the Heart Spectrum Blood Pressure Monitor on the arm detects the blood pressure and depicts the time-domain pressure wave. The time-domain pressure wave is converted to an energy-domain frequency wave via FFT (Figs 2 and 3). There are primary frequency peaks when the wave is converted via FFT. When observing abnormal frequency, the frequency peaks other than the primary frequency peaks are considered heart noises, and can be quantified as the heart index, as described below. We defined the first frequency region as the first heart rate frequency ± 0.5 frequency interval, the second frequency region as the second heart rate frequency ± 0.5 frequency interval, and the third frequency region as the third heart rate frequency ± 0.5 frequency interval. For example, if the first heart rate frequency is 60 beats per minute (1 Hz), then the first frequency region is 30 to 90 beats per min, the second frequency region is 90 to 150 beats per min, and the third frequency region is 150 to 210 beats per min, wherein the heart index I1 is the sum of noise in the first frequency region, the heart index I2 is the sum of noise in the second frequency region, and the heart index I3 is the sum of noise in the third frequency region. The heart index = I1 + I2 + I3. We defined the heart noise as the number of other spikes above 1/20 for each region. The scale factor of 1/20 was determined by removing the background noise from clinical pre-test results.

#### AF analysis

Measurements were obtained from each subject consecutively three times using method 1 (M1), method 2 (M2), and method 3 (M3).

M1 involved the following: standard blood pressure measurement was used to determine the heart index and was compared with the 12-lead ECG synchronously. M2 involved the following: from M1, the systolic and diastolic pressures were obtained and the mean arterial pressure (MAP) was calculated (Eq 1). MAP was then used as the constant pressure measurement to determine the heart index and was compared with the 12-lead ECG results at the same time.

MAP=[2×(Diastolic)+(Systolic)]3 (1)

M3 involved the following: a constant pressure (60 mmHg) was used to analyze the heart index and to compare it with the simultaneous 12-lead ECG results.

The sensitivity, specificity, PPV, and NPV of AF were analyzed via individual analysis and comprehensive analysis at the end of the trial.

The heart index criteria for judging AF were as follows: AF, (I1 + I2 + I3) ≥ 5.0 and I1 ≥ 2.0; and non-AF, (I1 + I2 + I3) <5.0.

#### Statistical analysis

Microsoft Excel statistical software (Microsoft, Redmond, WA, USA) was used for the analysis. The statistical analysis programs for sensitivity, specificity, PPV, and NPV are noted in the following list:

- Sensitivity = A / (A + C),
- Specificity = D / (B + D),
- PPV = A / (A + B), and
- NPV = D / (C + D),

where A, B, C, and D, represent true positive, false positive, false negative, and true negative of AF, respectively, based on 12-lead ECG.

The detailed Statistical Analysis Plan is given in S3 File.

## Results

The clinical trial involved 63 subjects; subjects’ statistics are shown in Table 1. One case of a 90-year-old man who had a pacemaker installed was excluded from the statistics. Using 12-lead ECG, the physicians identified atrial flutter in a 70-year-old man. Although atrial flutter is also a type of arrhythmia, the focus of this clinical trial was AF; therefore, this patient with atrial flutter was included in the non-AF statistics and discussion.

**Table 1:** Patient demographics.

	All	AF via 12-Lead ECG	Non-AF via 12-Lead ECG
**Patients, n**	62	29	33
**Age (y), median (range)**	67 (25–97)	78 (39–97)	55 (25–84)
**Male, n (%)**	35 (56%)	14 (48%)	21 (64%)

Abbreviations: AF = atrial fibrillation, ECG = electrocardiogram, n = number. The data from one patient was excluded because the patient had a pacemaker.

### Results according to M1 of the clinical trial and analysis

Of the 29 patients with AF, a 73-year-old man was falsely judged as not having AF on heart spectrum analysis. Conversely, of the 33 patients without AF, a 67-year-old man was falsely judged as having AF on heart spectrum analysis.

Ventricular premature complex (VPC) signal effects were also examined. A 48-year-old man with AF had two VPC signals, and a 92-year-old man with AF had one VPC signal that could be successfully identified as true positive on heart spectrum analysis. The test results were not affected by the VPC signals. Moreover, an 82-year-old man without AF had four VPC signals that were successfully identified as true negative on heart spectrum analysis. The test results were not affected by the VPC signals.

From these test results, we calculated the sensitivity, specificity, PPV, and NPV were 97%, 97%, 97%, and 97%, respectively.

### Results according to M2 of the clinical trial and analysis

Of the 29 patients with AF, an 81-year-old woman and a 92-year-old man with AF were falsely judged as not having AF on heart spectrum analysis. All 33 non-AF subjects were correctly identified as not having AF on heart spectrum analysis.

Regarding VPC signal effects, a 48-year-old man with AF had four VPC signals; these were successfully identified as true positive on heart spectrum analysis. The test results were not affected by the VPC signals. Moreover, an 82-year-old man without AF had five VPC signals, and a 67-year-old man without AF had one VPC signal; these were correctly identified as true negative on heart spectrum analysis. The test results were not affected by the VPC signals.

From the test results, we calculated that the sensitivity, specificity, PPV, and NPV were 90%, 100%, 100%, and 91%, respectively.

### Results according to M3 of a clinical trial and analysis

All 29 patients with AF were correctly identified on heart spectrum analysis as true positives. Of the 33 patients without AF, a 67-year-old man was falsely judged to have AF on heart spectrum analysis.

For the VPC signal effects, an 81-year-old woman with AF had one VPC signal, a 48-year-old man with AF had two VPC signals, and an 88-year-old woman with AF had one VPC signal, all of which were successfully identified as true positive on heart spectrum analysis. The test results were not affected by the VPC signals. Conversely, an 82-year-old man without AF had up to six VPC signals during approximately 10 s that were misjudged as AF on heart spectrum analysis; this was a false positive result.

From the test results, we calculated that the sensitivity, specificity, PPV, and NPV were 100%, 94%, 94%, and 100%, respectively (Table 2).

**Table 2:** Comparison of sensitivity, specificity, PPV, and NPV.

Method	Sensitivity (%)	Specificity (%)	PPV (%)	NPV (%)
**M1**	97	97	97	97
**M2**	90	100	100	91
**M3**	100	94	94	100

Abbreviations: PPV = positive predictive value, NPV = negative predictive value, M1 = method 1, M2 = method 2, M3 = method 3.

## Discussion

The purpose of this clinical trial was to compare the Heart Spectrum Blood Pressure Monitor and 12-lead ECG in verifying the diagnosis of patients with AF. Sensitivity and specificity are used to determine the accuracy of diagnostic tools. According to the analysis of the M1, M2, and M3 results, M3 was superior to the other methods. The test results showed a sensitivity of 100%, specificity of 94%, PPV of 94%, and NPV of 100%.

To confirm the diagnosis of AF, PPV is the most important measurement because PPV is the true positive ratio of the diagnosis. From this point of view, M2 is superior to other methods because the test results showed a sensitivity of 90%, specificity of 100%, PPV of 100%, and NPV of 91%.

**Fig 4:**
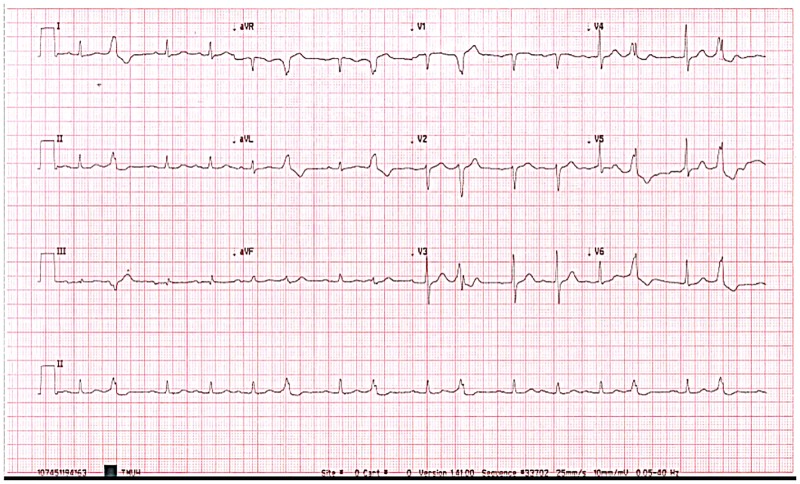
Twelve-lead ECG of the subject with six VPC signals.

From the results of the clinical trial for VPC statistics (Table 3), we determined that patients who had fewer than six VPC signals within 10 seconds did not produce false positives for AF interpretation between 12-lead ECG and blood pressure monitoring. Conversely, VPC signals greater than or equal to 6 within 10 seconds affected the AF interpretation. For example, there was an 82-year-old man without AF who had six VPC signals within approximately 10 s on the 12-lead ECG (Fig 4), and was falsely judged as having AF via the heart spectrum analysis. The reason for these false judgements may be that the main heartbeat peak of the subject was regular, and the number of VPC signals was up to six within 10 s and were judged as another regular frequency; therefore, the results were not compatible with the heart index criteria for judging AF. VPC is a very important topic of discussion. Although there were not many cases of more than six VPC signals for analysis, this should be an issue that is investigated in future trials.

**Table 3:** Demographics of patients with VPC signals.

Age (y)/Sex	Method	VPC Signals, n	12-Lead ECG, AF or non-AF	BPM with Spectrum, AF or non-AF
48/M	M1	2	AF	AF
	M2	4	AF	AF
	M3	2	AF	AF
67/M	M3	1	Non-AF	Non-AF
81/F	M2	1	AF	AF
82/M	M1	4	Non-AF	Non-AF
	M2	5	Non-AF	Non-AF
	M3	6*	Non-AF	AF
88/F	M3	1	AF	AF
92/M	M1	1	AF	AF

Abbreviations: VPC = ventricular premature complex, n = number, ECG = electrocardiogram, BPM = blood pressure monitoring, M = male, F = female, AF = atrial fibrillation.

*A VPC signal greater than or equal to six within 10 s led to misinterpretation of AF diagnosis.

Three measurement methods were used in this study: blood pressure (M1); MAP (M2), which was calculated from individual blood pressure as constant pressure; and a constant pressure of 60 mmHg (M3) to analyze the heart index and compare it with simultaneous 12-lead ECG.

Atrial flutter was identified using 12-lead ECG in a 70-year-old man. Although atrial flutter is a type of arrhythmia, the focus of this clinical trial was AF; therefore, the patient with atrial flutter was included in the non-AF group for statistical analysis. In future trials, other types of arrhythmia patterns should be included with more waveform analyses.

Users can determine and pre-diagnose the occurrence of AF using the Heart Spectrum Blood Pressure Monitor at home. Compared to using the general 12-lead ECG, which requires the users to visit the hospital for measurement and diagnosis by an examining physician, this application provides a more convenient and accurate tool for pre-diagnosis of AF. Patients can be treated with anticoagulants immediately after diagnosis to effectively reduce the risk of systemic embolization of non-valvular AF. Furthermore, justification for a home evaluation of AF includes the potential for decreased AF complications, such as strokes. However, AF is frequently asymptomatic; therefore, using the device during community screenings for AF is particularly beneficial to achieve early detection and treatment, especially for elderly individuals living alone. Further data collection will also facilitate future implementation of telecare. Using the devices could also prevent human error and help general practitioners and primary care physicians who have minimal experience reading an ECG.

A previous study also tested the use of home blood pressure monitors for AF diagnosis [20], where the device measured the last 10 pulse intervals during cuff deflation, and calculated the mean and standard deviation of the intervals for analysis. In that study, the number of beats was analyzed to detect AF based on the time domain. In the present study, we used the Heart Spectrum Blood Pressure Monitor to measure cardiovascular pressure change with FFT analysis to obtain the heart rate frequency based on frequency domain. The advantage of the frequency analysis was that we could clearly judge the normal and abnormal frequencies.

## Conclusions

We used three methods during this clinical trial, and the sensitivity, specificity, PPV, and NPV were all in the range of 90% to 100%. The results suggest that the Heart Spectrum Blood Pressure Monitor can be used for the diagnosis of AF. Through a simple measurement of blood pressure, users can detect the occurrence of AF at home and use this information as an early warning for further consultation with a physician. The device provides a convenient and effective filtering application compared to traditional methods that require visiting a physician for a 12-lead ECG.

### Future research

Through future clinical trials, we will be able to further analyze and explore big data, compare and analyze the different arrhythmia etiologies of various conditions, and further explore the conditions of patients with a heart index <5 (non-AF) for the differential diagnosis of diseases. In addition, telecare technology can be used by patients and healthy individuals to assess their heart function status, and maintain data for their own reference or for consultation with professional health care workers.

## Supporting information

S1 File Original Clinical Research Protocol.pdf—Original Clinical Research Protocol. (PDF)

S2 File Trial-protocol_20170504.doc—Clinical Trial Protocol. (DOC)

S3 File Statistical Analysis Plan_20170503.doc—Statistical Analysis Plan. (DOC)
