# Supplementary material for: Assessment of the clinical efficacy of the heart spectrum blood pressure monitor for diagnosis of atrial fibrillation: An unblinded clinical trial
Source: PLoS One. 2018 Jun 14;13(6):e0198852. doi: 10.1371/journal.pone.0198852 (PMC6001975; doi:10.1371/journal.pone.0198852)
Supplement: S1 File — (PDF) [file pone.0198852.s001.pdf]

臺北醫學大學暨附屬醫院聯合人體研究倫理委員會
TMU-Joint Institutional Review Board

試驗計畫名稱：評估心臟頻譜血壓計用於診斷心房顫動之臨床功效

試驗計畫版本/日期：_version1.0/20150505

試驗計畫書有任何修正，必須有修正編號及日期

計畫書編號：OSTAR 88
計畫書版本：version 1.0
計畫書版本日期：20150505

試驗委託廠商/機構及聯絡人

廠商/機構名稱：	源星生醫科技股份有限公司		
姓名：	洪健銘		
地址：	新北市新店區民權路 46-4 號 5 樓		
辦公室電話：	02-29182390	傳真：	02-29182683
電子郵件信箱：	sales@ostar.com.tw		

試驗計畫主持人

姓名：	高偉峰	單位：	台北醫學大學附設醫院
地址：	台北市信義區吳興街 252 號		
辦公室電話：	02-27372181#8107	傳真：	02-27366189
電子郵件信箱：	wfkao@hotmail.com.tw		

※主持人聲明※

本人負責執行此臨床試驗，依赫爾辛基宣言的精神，醫療器材優良臨床試驗準則及國內相關法令的規定，來落實執行此計畫。務求確保試驗對象之生命、健康、個人隱私及尊嚴。

計畫主持人姓名：高偉峰

簽章：高偉峰　　　　日期：2015.8.19

目 錄

1 摘要 1

2 試驗介紹 2

2.1 研究用產品/製品/器材介紹 2
2.2 臨床前試驗數據 3
2.3 先前的臨床試驗數據 3
2.4 風險/效益評估 4
2.5 法規依循 5
2.6 受試者族群介紹 5
2.7 參考文獻資料整理 7

3 試驗目的 9

4 試驗設計 9

4.1 主要試驗目的/次要試驗目的 10
4.2 試驗設計方式 10
4.3 試驗治療程序 11
4.4 試驗期間 13
4.5 停止試驗之條件 13
4.6 研究用產品的給予、使用及管理 13
4.7 試驗數據之收集方式 13

5 受試者的選擇及退出 14

5.1 受試者納入條件 14
5.2 受試者排除條件 14
5.3 受試者退出試驗的情況 14
5.4 試驗治療方法與處置流程 14
5.5 併用與禁用藥物/治療 15

6 療效評估 15

6.1 用以評估試驗療效之參數 15
6.2 評估方法及時間點 15

7 安全性評估 16

7.1 評估方法及時間點 16
7.2 不良事件之通報 16
7.3 不良事件之定義 16
7.4 不良事件追蹤 17

8 統計分析 17

8.1 統計方法 17
8.2 預定收納的受試者人數及分析族群的選擇 17
8.3 對於未採用資料的處理程序 18
8.4 違反計畫書所載之統計方法的報告流程 18

9 原始資料的直接檢視 18

10 品質管制 18

11 倫理考量 19

12 資料處理及保存 20

13 財務及保險 20

14 參考文獻 22

15 附錄 26

附錄一、赫爾辛基宣言 27

赫爾辛基宣言（DECLARATION OF HELSINKI） 27

附錄二、嚴重不良事件(SAE)/安全性報告作業程序 29

嚴重不良事件(SAE)/安全性報告作業程序 29

縮 寫 表

AF	Atrial Fibrillation
AFL	Atrial Flutter
CFR	Case Report Form
ECG	Electrocardiography
EPS	Electrophysiologic study
FFT	Fast Fourier Transformation
GCP	Good Clinical Practice
ICF	Informed Consent Form
ITT	Intent to Treat
MVR	Moderate Ventricular Response
PP	Per protocol
PAC	Premature Atrial Contraction
PVC	Premature Ventricular Contraction
PSVT	Paroxysmal Supraventricular Tachycardia
RVR	Rapid Ventricular Response
SAE	Serious Adverse Event
SVR	Slow Ventricular Response
VF	Ventricular Fibrillation
VT	Ventricular Tachycardia

1 摘要

隨著生活富裕與飲食習慣改變，加上生活步調變快、壓力增加，心律不整的病患與疾病的複雜度與日俱增，對心律不整治療專業需求也日益加重。許多心律不整表現是無症狀的，一旦有症狀則常以心悸或心跳暫停的感覺表現。嚴重的心律不整會導致頭暈、昏厥、呼吸困難或胸痛。即使大部分情況是良性的，有些心律不整，例如心房顫動卻會增加中風或心臟衰竭等併發症的風險，甚至導致心跳驟停休克、猝死。根據各大醫院逐年臨床病例觀察結果顯示，心臟血管疾病病例、心肌梗塞及中風病人顯著增加，且罹患年齡亦有下降趨勢。早年急性心肌梗塞在年輕人相當少見，但近年來卻案例頻傳。因此，如何及早發現心臟血管機能異常以及如何診斷是否具有危急性的心臟病變，以即時給予適當治療，成為相當重要課題。心律不整的診斷方法目前以十二導程心電圖及動態心電圖最為常見。在心電圖檢查中能記錄到心律不整的發作，進而判斷其心律不整的類別及嚴重度。“源星”心臟頻譜血壓計是利用心臟血管壓力變化(非統計模式)得到心臟跳動頻率雜訊及精確血壓資料的儀器。本產品可產生類似心電圖之波形，因此可應用於診斷心臟異常患者。為評估心臟頻譜血壓計用於診斷心房顫動之臨床功效，本試驗採開放方式(非盲性)進行臨床研究。受試者在簽署同意書後將同時進行心電圖與心臟頻譜檢查。測試期間試驗醫師將隨時觀察是否有無不良反應之發生。檢查結果將由兩位醫師進行判讀，最後藉由分析心臟頻譜血壓計鑑別診斷心律不整之靈敏度與特異性評估本產品之臨床功效。本試驗將於台北醫學大學附設醫院執行（單一臨床試驗中心），試驗期間大約一年，預估收錄 60-100 人參加。

2 試驗介紹

2.1 研究用產品/製品/器材介紹

- 產品名稱：心臟頻譜血壓計(Ostar P2)
- 用途：心臟頻譜血壓計是利用心臟血管壓力變化(非統計模式)得到心臟跳動頻率雜訊及精確血壓資料的儀器。藉由一般性的血壓量測檢查，除了得到收縮壓、舒張壓、脈搏等重要資料，同時運用數位訊號處理器計算出心臟雜訊，分別解析出心臟頻率區、辦膜區、心血管區，三區域之頻率。使用者可居家觀察心臟頻率變化，達到健康自主管理的目的。
- 原理：
  - ✧ 心臟頻譜血壓計是一種經手臂肱動脈，以頻率(frequency)為單位記錄血管壓力的生理活動，透過手臂上的壓力變化以快速傅立葉(Fast Fourier Transformation, FFT)轉換並記錄的診療技術。
  - ✧ 心臟竇房結產生起搏，推動血液由主動脈辦射出，傳輸血液至全身血管。藉由手臂或手腕血壓群波可量測血管壓力的變化，繪出血管壓力圖，再轉換成心臟頻譜圖後可計算心臟主動脈辦射血的變化。
  - ✧ 每次心跳從心臟射出血液，藉由手臂上的傳感器偵測血管壓力後經記錄裝置捕捉並放大描繪出時間壓力圖。由時間壓力圖轉成能量頻率圖，觀察是否有不正常頻率，不正常頻率即為心臟雜訊(Heart Noise)。心臟頻譜血壓計可以計算整個心臟泵血的頻率。
- 特色：
  - ✧ 心臟頻譜血壓計是測量和診斷異常心臟泵血節律的最好的方法。

- ✧ 可診斷心臟泵血輸出異常以及由於心肌壞死引起的心臟泵血輸出強度節律的改變。
- ✧ 由於心臟辦膜缺損會影響血液輸出狀況，心電圖無法看出，心臟頻譜血壓計可看出心臟辦膜其差異。
- ✧ 心臟頻譜血壓計量測可同時量測血壓及心臟頻譜。
- ✧ 可於醫生看診時量測量，不需更衣。
- ✧ 於家中遠距健康照護時，使用容易。

- 使用方法：請參照使用說明書。

2.2 臨床前試驗數據

本產品通過電性安全試驗(IEC 60601-1)以及電磁相容性試驗(IEC 60601-1-2)，安全無虞。在功能性測試部分，包括壓力量測準確度、環境性能、信號輸入與輸出、電源電壓變化的影響以及整體系統準確度等，皆符合法規規範。

2.3 先前的臨床試驗數據

本產品曾於國泰醫院進行「心臟雜訊在心血管疾病預警相關性之研究」。該研究之目的在利用血壓計結合脈動分析功能檢視心臟機能，藉以評估該產品在心臟異常偵測之準確率。

在201位使用者中，53位為心臟病患者，148位為非患有心臟疾病者。將心臟病患者大略歸類四項：(1)辦膜結構方面問題，泛指各種辦膜畸形及異常；(2)心肌病變，除了心肌病（cardiomyopathy）之外，在此將心肌炎（myocarditis）、心包炎（pericarditis）包含其中；(3)節律方面問題，屬心律不整，包括心房撲動（atrial flutter）、竇性心律不整、竇房傳導異常等；(4)缺血性心臟病，指冠狀動脈方面疾病，此為國內最多數的心臟病類型。

53 例心臟病患中，包括辦膜性疾病 13 例、心肌病變 12 例、心律不整 7 例以及冠狀動脈疾病 21 例。13 例辦膜性疾病之異常現象偵測率達 84.62%，加上疑似異常偵測率 7.69%，共達 92.31%。11 例心肌病變樣本中，異常現象偵測率 66.67%，加上疑似異常偵測率 25%，可測得之比例達 91.67%，亦屬於容易測得之狀況。心律不整樣本數共 7 例，其中疑似異常比例為 57.14%、異常現象偵測率為 28.57%，共 85.71%。冠狀動脈疾病之樣本數共 21 例，其中異常現象偵測率 38.10%，疑似異常偵測率 28.57%，總和 66.67%，遺漏率達 33.33%。

148 位非患有心臟疾病者中，非心臟病但患有高血壓者共 110 例，非心臟病且非高血壓者 38 例。高血壓族群異常現象偵測率 36.37%，較非高血壓者 18.42%高，亦即高血壓病患之心肌收縮頻率疑似異常或異常之比例較一般人高。事實上高血壓族群為心血管疾病罹患之高危險群之一，其中少數已有徵兆。

該研究利用血壓偵測器紀錄之訊號進行研究，經過適當擷取訊號，發現在正規化的能量頻譜圖中，具有異於正常脈動分析結果之特徵。再定義量化之指標『心臟指數（heart index）』後，由程式自動求出的此數值，可作為機能狀況之參考指標，藉以觀察心搏跳動是否存在異常頻率，亦可視為量測血壓之外的附加價值。此理論已於國泰醫院心臟內科做過初步驗證，與醫師之診斷符合率約達八成。

2.4 風險/效益評估

心房顫動（Atrial Fibrillation, AF）是消耗社會成本很重的一種心律不整疾病。它可發生在一般似同健康的人，也可附加於其他心臟血管疾病之上，心房顫動除了讓病人感覺到心悸及不舒服之外，亦可引起中風及心力衰竭。“源星”心臟頻譜血壓計是利用心臟血管壓力變化(非統計模式)

得到心臟跳動頻率雜訊及精確血壓資料的儀器。藉由不同時間的量測得知心臟及血壓在不同環境及狀況下之變化，因此可作為心臟健康自主管理的依據以及預防心肌梗塞之工具。此外，心臟頻譜血壓計可以評估心臟泵血能力以及瓣膜狀況，相較於心電圖，具有多元的功能。再者，由於進行心電圖(Electrocardiography, ECG) 檢查時需脫去上衣且需由專業人員操作，相較於心臟頻譜儀，本產品在使用上更為便利。在風險評估方面，心臟頻譜儀並不會產生任何的副作用與併發症，且亦通過電性安全相關測試，對人體不會造成任何的傷害。因此預期心臟頻譜血壓計所帶來之效益應可明顯地可增進受試者的福祉。

2.5 法規依循

- 本試驗將遵守經審查核准之試驗計畫書及其修正書之內容進行試驗。
- 本試驗將遵守「赫爾辛基宣言（Declaration of Helsinki）」(附錄一)、「醫療器材優良臨床試驗基準（Good Clinical Practice, GCP）」以及臺北醫學大學暨附屬醫院聯合人體研究倫理委員會之規範執行臨床試驗。

2.6 *受試者族群介紹*(1-5)

心臟節律不整，簡稱為心律不整(Cardiac Arrhythmia)，是指心臟電傳導系統異常所引起的各種症狀，包含心跳不規則、過快、或過慢的表現總稱。心律不整可能發生在心房，也有可能在心室。有些心律不整是原發性的，也就是心臟本身沒有結構上或功能上的異常，單純是心臟電路系統的問題。有些心律不整是因其他心臟疾病引起，如冠狀動脈疾病、風濕性心臟病、瓣膜性心臟病或高血壓心臟病等引起。

在正常人身上有時候也可能出現不規則跳動，這可以是正常的現象，但需要經過心電圖的確認。正常人的心跳速率會受到許多因素的影響而改變，例如運動時心跳會加快、自主神經興奮與否、咖啡或茶的刺激、發燒、緊張、壓力過大、疼痛、缺氧、貧血或肺栓塞、低血壓及藥物，均可能使心跳速率或節律有所改變。另外一種是心臟內多出一或兩個以上的節律點，或者多出一個兩條以上傳導路徑，使得心跳節律與速率均異於平常。

心律不整分為四大類：期外收縮、心室上心搏過速、心室心律不整及心搏過緩。期外收縮包括心房期外收縮(Premature Atrial Contraction, PAC)及心室期外收縮(Premature Ventricular Contraction, PVC)。心室上心搏過速包括心房顫動(AF)、心房撲動(Atrial Flutter, AFL)、以及陣發性心室上心搏過速(Paroxysmal Supraventricular Tachycardia, PSVT)。心室心律不整包括心室顫動(Ventricular Fibrillation, VF)和心室心搏過速(Ventricular Tachycardia, VT)。許多心律不整表現是無症狀的，一旦有症狀則常以心悸或心跳暫停的感覺表現。嚴重的心律不整會導致頭暈、昏厥、呼吸困難或胸痛。即使大部分情況是良性的，有些心律不整卻會增加中風或心臟衰竭等併發症的風險，甚至導致心跳驟停、休克、猝死。

心房顫動(AF)是臨床上常見的一種心律不整。一般而言，AF 的發生和心房的退化病變有關。因此，年齡越大，發生率越高。根據統計，65 歲上下的族群約少於 5-6%有 AF，但在 80 歲以上的族群約 8%以上有 AF 且男性比起女性有較高的發生率 [1,2]。此外患有高血壓、心衰竭、瓣膜性心臟病（尤其是風濕性）、糖尿病或是甲狀腺亢進的病人也較容易發生心房顫動 [3,4]。當心房顫動發生時，對於心臟的影響大致有兩大類：第一，因為心跳速率忽快忽慢且不規律，心臟的血液輸出量便會減少，病人血壓就有可能下降，而出現心悸、胸悶、呼吸困難、喘、頭暈

等症狀。反之，當心跳數變得很快時，心臟血液輸出量除減少外，久而久之心臟功能也會惡化，造成心臟衰竭之現象。第二，心房顫動發生時，因心房組織快速不正常放電，心房便無法正常且有效的收縮，取而代之的是快速地顫動，導致血液無法有效且正常的移動，因而容易於左心房產生血栓，當血栓隨著血流流出心臟，便容易造成器官的栓塞，如栓塞於腦部便就是腦中風。根據流行病學的調查發現，心房顫動的病人發生腦中風的機會是正常人的四倍以上 [5]，所以有心房顫動的病人，就必須服用藥物來預防腦中風的發生。

2.7 參考文獻資料整理

在全世界人口逐漸老化的趨勢下，罹患心房顫動的人數不斷的攀昇。心房顫動的病患可能引發的併發症包括心臟衰竭、心絞痛、血壓降低以及中風等。根據研究報告指出，患有 AF 的病患發生死亡的風險是正常人的 2 倍，發生中風的風險是正常人的 4-5 倍，心臟衰竭是正常人的 10 倍 [5-9]。造成心房顫動的危險因子包括：年齡、高血壓、糖尿病、心臟辦膜疾病、心衰竭、肥胖、睡眠呼吸中止症與大量喝酒等。隨著心房顫動疾病的進展，心房肌肉組織的病變逐漸加重，心房顫動發作的時間由「陣發性」逐漸成為「持續性」及「慢性」，心跳的刺激變成不規則的節律，使得心臟功能進一步惡化。

由於心律不整的分類眾多，因此正確的診斷是有其必要性。經由詳細病史的詢問、相關的檢查、十二導程心電圖及動態心電圖的紀錄 [10-12]，可以診斷大多數心律不整的案例。然而，就目前全球醫療院所使用之設備技術而言，對於初期及中期心臟病之診斷仍存盲點，有待突破。目前有關心臟血管生理功能之研究和檢查，大致有以下幾種方向 [13,14]：

(1). 心電圖：心電圖是記錄心臟組織電壓變化的一個圖形，原理是利用心臟傳導系統發出電波，興奮整個心臟肌肉纖維而產生收縮。電波的產生及傳導，皆會產生微弱的電流分佈全身，若將心電圖記錄器的電極連接到身上不同的部位，就可描出「心電圖」。然而心電圖的使用，即使紀錄運動心電圖，對於構造方面的心臟問題仍不易判斷。

(2). 心臟電氣生理檢查(Electrophysiologic study, EPS)：利用心導管的技術，置放多條電極導管到心臟內，記錄心臟內心電圖的變化及心律傳導的徑路，並經由電極導管刺激心臟來誘發出病人潛在可能發作的種種心律不整。也就是把病人平時發作的陣發性心跳過快或心跳過慢的情形，利用電氣生理學檢查方法，讓病人發作，並由發作時的心導管記錄正確診斷出心律不整的致病機轉，並加以正確的治療。

(3). 壓力體積環路圖(P-Vloop)：藉由量測左心室之收縮末期壓(end-systolic pressure)、舒張末期壓(end-diastolic pressure)、心臟搏動容積(stroke volume)等，求出左心室之壓力與體積環路圖，以分析判斷心臟作功(work)之大小與變化。由於複雜與系統龐大之缺點，近幾年較少相關研究。

(4). 血壓波形：心室肌肉的收縮與舒張，是血壓脈動發生的主要原因。血壓與血流均以脈動的方式在血管系統內傳遞，不過兩組脈動波並不一致。血壓波大致沿血管壁傳遞，血流波則侷限在血管內緩慢移動。在整個血壓的傳遞途徑上，血壓波形受到諸多影響而產生變化。透過對血壓波形變化的分析與研究，可以了解許多心臟血管的特性。此方面研究之延伸，以王唯工博士『血壓波與體內器官共振現象之探討』系列研究最為著名，推測血壓波形的傳導與體內器官

共振機制循環系統之密切關係。目標在於利用共振聲波達到循環疾病調整、治療功效。

(5). 醫學影像：影像處理技術發展日新月異，配合偵測儀器，有助於精確解讀生理結構，協助判讀非侵入式偵測儀器所得之訊息。例如超音波量測技術配合彩色三維顯像、電腦斷層掃描、核磁共振等，在臨床診斷上有著實的貢獻。唯此研究儀器昂貴，適用於大型醫療院所，難以朝居家保健的應用方向發展。

儘管對於心臟血管疾病之探討與研究不少，但其發展過程與成果應用皆偏重於大型醫療院所方能負擔之儀器設備。然而一般民眾或患者有機會使用到大型昂貴醫療儀器時，往往已到病症足以危害生命之程度，因此耗費醫療系統資源極龐大。基於此趨勢，朝『預防』的角度進行方法及儀器研究發展，是對人們的健康維護及即時警告十分有幫助的。

3 試驗目的

心律不整的診斷方法目前以十二導程心電圖及動態心電圖最為常見。在心電圖檢查中能記錄到心律不整的發作，進而判斷其心律不整的類別及嚴重度。心臟頻譜血壓計是利用心臟血管壓力變化(非統計模式)得到心臟跳動頻率雜訊及精確血壓資料的儀器。本產品可產生類似心電圖之波形，因此可應用於診斷心臟異常患者。本試驗主要是希望藉由比較本產品與心電圖的診斷效果，評估心臟頻譜血壓計用於診斷心房顫動之臨床功效。

4 試驗設計[15-22]

此試驗採開放方式(非盲性)進行臨床研究。受試者在簽署同意書後將同時進行心電圖(spacelabs sl3800)與心臟頻譜檢查。測試期間試驗醫師將隨時觀察是否有無不良反應之發生。檢查結果將由兩位醫師進行判讀，最後

藉由分析心臟頻譜血壓計鑑別診斷心房顫動之靈敏度(sensitivity)與特異性(specificity)，評估本產品之臨床功效。

4.1 主要試驗目的/次要試驗目的

- 主要療效指標：分析心臟頻譜血壓計鑑別診斷心房顫動之靈敏度(sensitivity)與特異性(specificity)。
- 次要療效指標：
  - ➢ 試驗中引發不良反應之比例
  - ➢ 比較心電圖與心臟頻譜血壓計之診斷確診率。
  - ➢ 心臟頻譜血壓計無法判讀之比例。

4.2 試驗設計方式

(1).	■對照：	■類似產品
		□其他
	□非對照：	
(2).	盲性：	■開放 □評估者盲性 □單盲 □雙盲 □雙虛擬 □其他
(3).	隨機分派:	□是 ■無
(4).	■平行 □交叉 □其他	
(5).	處置期間:	單次檢測
(6).	劑量調整：	□強制性 □選擇性 ■無
(7).	□多國多中心 □台灣多中心 ■台灣單中心	

試驗時程表

期間	篩選期	治療期
納入條件	x	
排除條件	x	
受試者同意書簽署		
病史問診	x	
生命徵象測量	x	
治療期間用藥情形		x
心電圖與心臟頻譜血壓計檢查		x
結果判讀		x
不良反應		x

4.3 試驗治療程序

尋求合適的研究對象，並向受試者解釋試驗的步驟、風險以及預期效益等資訊。讓受試者在充分的考慮時間下，簽署受試者同意書後，始得進入試驗。於篩選期間，研究醫師除了須確認受試者符合納入條件與排除條件，亦應針對身體狀況和病史作進一步的檢查和問診。試驗期間，受試者將同時以心電圖(spacelabs sl3800)與心臟頻譜血壓計偵測心臟運作之狀況，其後再由兩位醫師進行鑑別診斷，並將結果紀錄於個案報告表中。

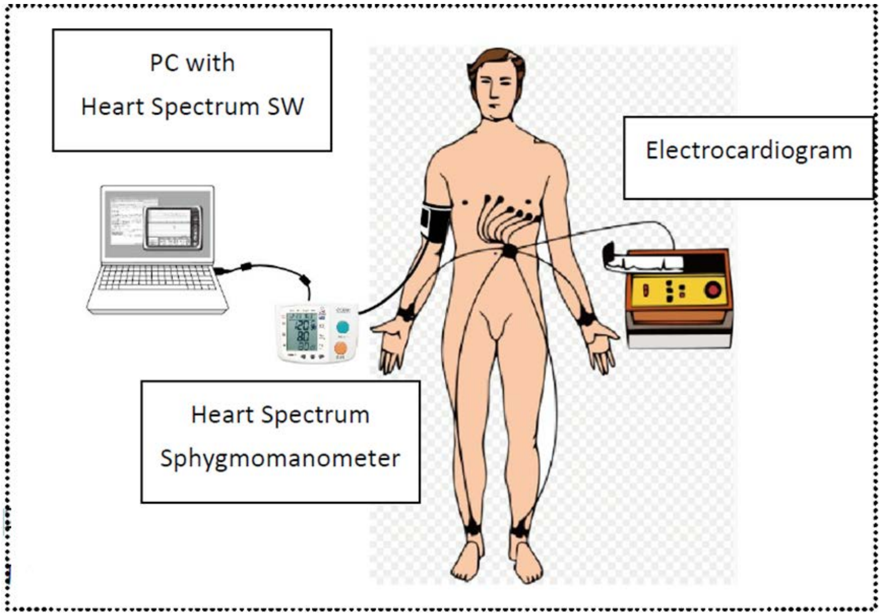

- 安裝心電圖貼片於受試者身體待測部位，並安裝血壓壓脈帶於該受試者手臂。
- 連接心臟頻譜血壓計與電腦。
- 執行電腦上的心臟頻譜軟體。
- 開始進行心臟頻譜血壓計量以及心電圖量測。
  i. 點選或按壓軟體上或心臟頻譜血壓計上"開始量測"按鈕；
  ii. 心臟頻譜血壓計打氣至 180 毫米汞柱(mmHg)；
  iii. 心臟頻譜血壓計洩氣過程並同時進行量測；
  iv. 與心臟頻譜血壓計量測同時，由專業醫師或醫護人員同步讀取並判斷心電圖量測結果；
  v. 同步量測過程約 10~30 秒完成；
  vi. 該心臟頻譜血壓計軟體介面將繪出心律圖形並經由快速傅立葉(FFT)分析計算心臟頻譜結果。
- 經由快速傅立葉計算心臟頻譜比對心電圖，臨床測試結果經由專業醫師進行判斷。

4.4 試驗期間

本試驗執行期間為 104 年 11 月 1 日至 105 年 10 月 31 日。

4.5 停止試驗之條件

試驗委託者或試驗主持人皆可因下列理由終止試驗，但不限於以下幾點：

- 研究者不能遵守試驗計畫書或醫療器材優良臨床試驗基準（Good Clinical Practice，GCP）。
- 安全方面的考慮。
- 有足夠的資料證實試驗器材缺乏療效。
- 研究者招募不到足夠的受試者。

4.6 研究用產品的給予、使用及管理

- 研究人員將確保收到的所有試驗器材都有庫存記錄，並在研究過程中清點。
- 試驗器材的使用須嚴格遵守試驗計畫書和使用說明書上的內容。
- 試驗器材的管理由指定之研究人員負責，且存放在只有被授權人才可以打開或者上鎖的櫃子裏，並在規定的環境條件下儲存。

4.7 試驗數據之收集方式

- 所有受試者試驗相關資訊將被記載在個案報告表上（Case Report Form, CRF），包括受試者基本資料、問診資料、測試結果以及受試期間所產生的任何徵象或症狀。每位加入試驗的受試者都需完成個案報告表，且完成的個案報告表需經試驗計畫監測者驗證與原始資料上無異後，才可進行後續資料處裡工作。
- 試驗主持人須持有一份適合且具資格的臨床研究人員名單，這些人須經過書面授權方可填寫或修改個案報告表。試驗主持人在確認或

檢查個案報告表上的資料後，須在個案報告表上明顯處簽上名字及日期。

5 受試者的選擇及退出

5.1 受試者納入條件

- 受試者年齡 20 歲以上。
- 受試者或法定代理人已瞭解試驗內容並且願意簽署受試者同意書。
- 受試者具備以下任何一項條件：
  - ✧ 以心電圖確診為 AF 之受試者。
  - ✧ 沒有 AF 之健康受試者。

5.2 受試者排除條件

- 測試期間會暴露在高頻手術設備者。
- 裝有心臟節律器或植入式去顫器之患者。
- 懷孕婦女。
- 無法遵守試驗計畫書要求之受試者。

5.3 受試者退出試驗的情況

- 嚴重不良反應
- 發生危急之臨床狀況，需以其他方式處置者。
- 未能遵守試驗規定
- 撤回同意

5.4 試驗治療方法與處置流程

尋求合適的研究對象，並向受試者解釋試驗的步驟、風險以及預期效益等資訊。讓受試者在充分的考慮時間下，簽署受試者同意書後，始得進入試驗。於篩選期間，研究醫師除了須確認受試者符合納入條件與排除

條件，亦應針對身體狀況和病史作進一步的檢查和問診。試驗期間，受試者將同時以心電圖(spacelabs sl3800)與“源星”心臟頻譜血壓計偵測心臟運作之狀況，其後再由兩位醫師進行鑑別診斷，並將結果紀錄於個案報告表中。

5.5 併用與禁用藥物/治療

- 試驗前或試驗期間不得裝置心臟節律器或植入式去顫器。

6 療效評估

6.1 用以評估試驗療效之參數

本試驗主要是藉由與心電圖檢查結果之比較，評估心臟頻譜血壓計用於診斷心房顫動之臨床功效。一般來說，利用心電圖判讀心房顫動之準則與方法如下：

- 不規則的心室心律(irregular ventricular rhythm)
- 沒明顯 P 波(亂抖)，R-R 不規則
- 有 3 種不同的心室反應(ventricular response)
  - ✧ 心房顫動合併慢速心室反應(Af with SVR (slow ventricular response))：心跳<60/分
  - ✧ 心房顫動合併適中心室反應(Af with MVR (moderate ventricular response))：心跳 >60/分, <100/分
  - ✧ 心房顫動合併快速心室反應(Af with RVR (rapid ventricular response))：心跳>100/分

6.2 評估方法及時間點

測試結束後由試驗醫師進行判讀並將結果紀錄於個案報告表中。

7 安全性評估

7.1 評估方法及時間點

於試驗期間所產生的任何不良反應，計算各症狀發生之百分比。

7.2 不良事件之通報

- 受試者發生任何嚴重不良事件，試驗主持人應在 24 小時內通知試驗委託者。
- 試驗主持人獲知死亡或危及生命之嚴重不良事件，應於獲知日起 7 日內向臺北醫學大學暨附屬醫院聯合人體研究倫理委員會報告，15 日內提出書面報告。有關通報流程與相關書面報告請參考臺北醫學大學暨附屬醫院聯合人體研究倫理委員會所訂定之「嚴重不良事件(SAE)/安全性報告作業程序」(附錄二)。
- 試驗主持人或試驗委託者獲知死亡或危及生命之嚴重不良事件，應於獲知日起 7 日內通報全國藥物不良反應通報中心，並在獲知日起 15 日內提供詳細書面資料。其他嚴重不良事件，應於獲知日起 15 日內通報全國藥物不良反應通報中心。

7.3 不良事件之定義

(1). 不良事件之定義

受試者參加試驗後所發生之任何不良情況。此項不良情況與試驗醫療器材不以具有因果關係為必要。

(2). 嚴重不良事件（Serious Adverse Event, SAE）之定義

係指因使用試驗醫療器材致生下列各款情形之一者：

- 死亡。
- 危及生命。
- 造成永久性殘疾。

- 胎嬰兒先天性畸形。
- 導致病人住院或延長病人住院時間。
- 其他可能導致永久性傷害需做處置者。

7.4 不良事件追蹤

- 任何不良事件之發生，試驗主持人應提供受試者充分醫療照護。
- 所有不良事件都應當追蹤紀錄，並依據相關規定通報，直到得到妥善解決或病情穩定為止。

8 統計分析

8.1 統計方法

於試驗結束後分析心臟頻譜血壓計鑑別診斷心房顫動之靈敏度(sensitivity)與特異性(specificity)。

8.2 預定收納的受試者人數及分析族群的選擇

- 預定收納的受試者人數：本臨床研究預計在台北醫學大學附設醫院執行（單一臨床試驗中心）。預計招募 60-100 位受試者以做為評估心臟頻譜血壓計臨床功效之可行性試驗(feasibility study)。
- 分析群體
  - ➢ 意圖治療的分析群體（Intent to Treat, ITT）：將至少接受過一次治療，且至少接受一次療效指標評估的受試者，定義為 ITT。
  - ➢ 依計畫書的分析群體（Per protocol, PP）：完成所有試驗流程的研究案例定義為 PP。
  - ➢ 安全性評估群體：分析群體以 ITT 為主。
  - ➢ 療效性評估群體：主要療效指標的分析是以 ITT 分析群體為主，PP 分析群體的結果亦會呈現，以評估 ITT 與 PP 分析結果是否一

致。

8.3 對於未採用資料的處理程序

當測試結果無法作為醫師鑑別診斷心臟運作之狀況時，此數據將不會納入療效評估的統計分析中。對於未採用的資料，試驗醫師必須分析引發該狀況的可能因素並紀錄於個案報告表中。

8.4 違反計畫書所載之統計方法的報告流程

任何偏離原定統計設計之任何事項需有正當之理由，且必須於最終報告中敘述與解釋。

9 原始資料的直接檢視

- 有關試驗委託者之監測、稽核，人體試驗委員會之檢閱，或主管機關之查核，試驗主持人或試驗機構應給予其直接視察原始數據及文件之權利。
- 試驗主持人有義務告知受試者並獲得受試者同意，才可讓相關人員接觸個案的試驗相關資料，且須在不違反受試者資料保密原則下才可進行。

10 品質管制

- 為確保受試者之權利及福祉受到保護，監測者必須確認受試者於參與試驗前皆已簽署受試者同意書，且確認相關人員及試驗儀器可適當、安全及正確地執行試驗。
- 在臨床試驗執行期間，監測者將確認試驗主持人是否遵守經審查核准之試驗計畫書及其修正書；報告受試者之收納進度；試驗器材的使用記錄；核對個案報告表（CRF）之登錄、原始資料、檔案以及所有不良事件是否均已依規定通報。監測頻率為 4 週執行一次，試驗單位應

將監測日期紀錄於《監測記錄表》。監測過程中，相關的研究人員必須在場，與監測者討論在核對 CRF 與原始記錄過程中發現的問題。另外，試驗機構必須提供一個可供監測者閱讀相關研究文件或檔案的場所。

- 為確保每位受試者的臨床資料品質，資料管理人員需檢查個案資料是否一致或是否有遺漏的地方。此外還需注意資料是否遵照計畫書和醫療器材優良臨床試驗基準。

11 倫理考量

- 本研究未涉及安慰劑對照的倫理學顧慮。
- 本產品已通過電性安全測試，對於受試者的安全性已作了審慎的評估與考量。
- 本試驗將遵守「赫爾辛基宣言（Declaration of Helsinki）」、「醫療器材優良臨床試驗基準（GCP）」以及「臺北醫學大學暨附屬醫院聯合人體研究倫理委員會」之規範執行臨床試驗。
- 本計畫書經「臺北醫學大學暨附屬醫院聯合人體研究倫理委員會」審核通過後方可執行。必要時，試驗主持人需向「臺北醫學大學聯合人體研究倫理委員會」報告試驗執行進度。
- 試驗主持人應詳實告知受試者試驗之目的和方法、預期效果、發生副作用與危險的可能性等資訊，並取得簽署之受試者同意書後，方可執行試驗。
- 本試驗之設計以保護受試者之權益為依歸，如試驗器材危害受試者之健康狀況時，當以其他治療方式處置。

12 資料處理及保存

(1). 臨床試驗相關資料將由指定之研究人員處理保存，電腦檔案會以加密處理，相關文件亦會存放在上鎖的櫃子中，鑰匙由研究人員保管。

(2). 試驗主持人及研究人員需有系統地集中管理臨床研究相關文件，方便試驗委託者或其他授權者隨時檢閱。臨床相關文件需包含：

- 個案資料夾須包含完整個案報告表、受試者同意書及其他相關資料。
- 研究資料須包含各版次計畫書、所有與試驗委託者或人體試驗委員會往來的信函及其他相關資料。
- 試驗器材使用記錄表及所有相關文件。

(3). 沒有試驗主持人和試驗委託者的同意，不能銷毀任何研究文件。研究人員或研究機構應採取措施以防止這些檔案或文件被提前銷毀。

(4). 如果負責的研究人員因退休、調離或其他原因不再承擔保存研究記錄的職責，必須將此監管任務交付給另一個願意承擔此項責任的人員，且以書面通知試驗委託者此新負責人的姓名和聯繫地址。

(5). 試驗主持人需堅守受試者資料保密原則，因此個案報告表或其他提供給試驗委託者的文件，只能以姓名縮寫或受試者編號來識別受試者。不提供給試驗委託者的文件（例如：已簽署之受試者同意書），須由試驗主持人嚴格保密管理。

13 財務及保險

- 本試驗所有費用支出完全由試驗委託者提供，受試者無須支付任何金錢。
- 試驗委託者須承擔因試驗器材所引起的醫療傷害責任，但不需要承擔

因醫師治療不當或疏忽所導致的醫療傷害責任。

- 若依臨床試驗計畫執行發生不良反應或傷害，將提供受試者專業醫療照顧及醫療諮詢。但本計畫書上所記載之不良反應，或這些不良反應所造成之預期傷害，將不予賠償或補償。

14 參考文獻

(1). Feinberg WM, Blackshear JL, Laupacis A, Kronmal R, Hart RG. Prevalence, age distribution, and gender of patients with atrial fibrillation. Analysis and implications. *Arch Intern Med*. 1995 Mar 13;155(5):469-73.

(2). Pérula-de-Torres LA, Martínez-Adell MA, González-Blanco M, Baena-Díez JM, Martín-Rioboó E, Parras-Rejano JM, González-Lama J, Martín-Alvarez R, Ruiz-Moral R, Fernández-García JÁ, Pérez-Díaz M, Ruiz-de-Castroviejo J, Pérula-de-Torres C, Valero-Martín A, Roldán-Villalobos A, Criado-Larumbe M, Burdoy-Joaquín E, Coma-Solé M, Cervera-León M, Cuixart-Costa L, Collaborative Group DOFA-AP: Opportunistic detection of atrial fibrillation in subjects aged 65 years or older in primary care: a randomised clinical trial of efficacy. DOFA-AP study protocol. *BMC Fam Pract*. 2012, 13:13–106.

(3). Stewart S, Hart CL, Hole DJ, McMurray JJ. A population-based study of the long-term risks associated with atrial fibrillation: 20- year follow-up of the Renfrew/Paisley study. *Am J Med.* 2002;113: 359-64.

(4). Benjamin EJ, Levy D, Vaziri SM, D’Agostino RB, Belanger AJ, Wolf PA. Independent risk factors for atrial fibrillation in a population-based cohort. The Framingham Heart Study. *JAMA* 1994; 271:840-4

(5). Wolf PA, Abbott RD, Kannel WB. Atrial fibrillation as an independent risk factor for stroke: the Framingham Study. *Stroke*. 1991 Aug; 22(8):983-8.

(6). Benjamin EJ, Wolf PA, D'Agostino RB, Silbershatz H, Kannel WB, Levy D. Impact of atrial fibrillation on the risk of death: the Framingham Heart Study. Circulation. 1998 Sep 8;98(10):946-52.

(7). Conen D, Chae CU, Glynn RJ, Tedrow UB, Everett BM, Buring JE, Albert CM. Risk of death and cardiovascular events in initially healthy women with new-onset atrial fibrillation. JAMA. 2011 May 25;305(20):2080-7.

(8). Wang TJ, Larson MG, Levy D, Vasan RS, Leip EP, Wolf PA, D'Agostino RB, Murabito JM, Kannel WB, Benjamin EJ. Temporal relations of atrial fibrillation and congestive heart failure and their joint influence on mortality: theFramingham Heart Study. Circulation. 2003 Jun 17;107(23):2920-5.

(9). Conen D, Osswald S, Albert CM. Epidemiology of atrial fibrillation. Swiss Med Wkly. 2009;139(25–26):346–52.

(10). Fung E, Järvelin MR, Doshi RN, Shinbane JS, Carlson SK, Grazette LP, Chang PM, Sangha RS, Huikuri HV, Peters NS. Electrocardiographic patch devices and contemporary wireless cardiac monitoring. Front Physiol. 2015 May 27;6:149.

(11). Camm AJ, et al. 2012 focused update of the ESC Guidelines for the management of atrial fibrillation: an update of the 2010 ESC Guidelines for the management of atrial fibrillation. Developed with the special contribution of the European Heart Rhythm Association. Eur Heart J. 2012;33(21):2719–47.

(12). Rosero SZ, Kutyifa V, Olshansky B, Zareba W. Ambulatory ECG monitoring in atrial fibrillation management. *Prog Cardiovasc Dis*. 2013 Sep-Oct;56(2):143-52.

(13). Andrade JG, Krahn AD. Is an implantable cardiac monitor the standard of care for determining the success of atrial fibrillation ablation? J Cardiovasc Electrophysiol. 2013 Oct;24(10):1083-5.

(14). Eitel C, Husser D, Hindricks G, Frühauf M, Hilbert S, Arya A, Gaspar T, Wetzel U, Bollmann A, Piorkowski C. Performance of an implantable automatic atrial fibrillation detection device: impact of software adjustments and relevance of manual episode analysis. *Europace*. 2011 Apr;13(4):480-5.

(15). Barrett PM, Komatireddy R, Haaser S, Topol S, Sheard J, Encinas J, Fought AJ, Topol EJ. Comparison of 24-hour Holter monitoring with 14-day novel adhesive patch electrocardiographic monitoring. *Am J Med*. 2014 Jan;127(1):95.e11-7.

(16). Home Monitoring for Atrial Fibrillation Using a Microlife Blood Pressure Monitor (TRIPPS). ClinicalTrials.gov Identifier: NCT00861354

(17). Six Lead Identification of Atrial Fibrillation (SL-AF). ClinicalTrials.gov Identifier: NCT02124629

(18). Ajijola OA, Boyle NG, Shivkumar K. Detecting and monitoring arrhythmia recurrence following catheter ablation of atrial fibrillation. *Front Physiol*. 2015 Mar 27;6:90.

(19). Barrett PM, Komatireddy R, Haaser S, Topol S, Sheard J, Encinas J, Fought AJ, Topol EJ. Comparison of 24-hour Holter monitoring with 14-day novel adhesive patch electrocardiographic monitoring. *Am J Med.* 2014 Jan;127(1):95.e11-7.

(20). Higgins P, MacFarlane PW, Dawson J, McInnes GT, Langhorne P, Lees KR. Noninvasive cardiac event monitoring to detect atrial fibrillation after ischemic stroke: a randomized, controlled trial. *Stroke.* 2013 Sep;44(9):2525-31.

(21). Vaes B, Stalpaert S, Tavernier K, Thaels B, Lapeire D, Mullens W, Degryse J. The diagnostic accuracy of the MyDiagnostick to detect atrial fibrillation in primary care. *BMC Fam Pract.* 2014 Jun 9;15:113.

(22). Tieleman RG, Plantinga Y, Rinkes D, Bartels GL, Posma JL, Cator R, Hofman C, Houben RP. Validation and clinical use of a novel diagnostic device for screening of atrial fibrillation. *Europace*. 2014 Sep;16(9):1291-5.

15 附錄

附錄一、赫爾辛基宣言

赫爾辛基宣言（Declaration of Helsinki）

2000 年中文版

甲.引言

1. 世界醫學會制定赫爾辛基宣言，作為醫師及醫學研究人員在人體試驗時之倫理指導原則。而所謂人體試驗之對象即包涵任何可辨識之人體組織或資料。
2. 醫師之職責在促進及維護人類之健康，其專業知識及良知應奉獻於此一使命。
3. 世界醫學會之日內瓦宣言（Declaration of Geneva）中，規範醫師必須以“病患之福祉為首要之考量”，而國際醫療倫理規章（International Code of Medical Ethics）亦宣示“在實施任何可能危及病患身心之醫療措施時，醫師應以病患之福祉為唯一之考慮。”
4. 醫學之進步奠基於科學研究，而此研究終究必須有部份仰賴以人為受試驗者。
5. 在進行有關人體試驗之醫學研究時，應將受試驗者之利益置於科學及社會利益之上。
6. 進行人體醫學實驗之首要目的，在於改進各種預防、診斷及治療之方法，及增進對於疾病成因之瞭解。對於目前已知最有效之預防、診斷及治療之方法，也應不斷地以研究來檢證其效果，效率，可行性，及品質。
7. 在當前的醫療行為及醫學研究中，大多數的預防、診斷及治療程序都涉及一定的危險與醫療責任。
8. 醫學研究之倫理標準，應以尊重生命，維護人類之健康及利益為依歸。對於較易受傷之受測族群必須特別加以保護。經濟弱勢及醫療資源匱乏之族群的特別需求也應加以關注。對於無法自行同意或拒絕研究的人、對於可能在脅迫下行使同意的人、對於那些無法因研究而親身受惠的人、及那些同時接受研究和醫療照護的人，也應特別關注。
9. 試驗主持人應注意該國與人體試驗有關之倫理、法律、及主管機關相關規定及適用的國際法規。任何國家之倫理、法律、條例之制定，皆不應減損或忽視本宣言對受試驗者所宣示之保障。

乙.醫學研究之基本原則

10. 醫學研究中，醫師之職責是在於保障受試驗者之生命、健康、個人隱私及尊嚴。
11. 任何涉及人體試驗之醫學研究，必須依循普遍接受之科學原則，並奠基於對科學文獻之徹底瞭解，相關資訊之掌握，及適當的研究數據及動物實驗。
12. 對於可能影響環境之研究都必須謹慎進行，而實驗動物之福祉也應予以尊重。
13. 在實驗計劃中，有關人體試驗的每一個實驗步驟，皆應清楚陳述其實驗之設計與執行。此試驗計畫書必須交由一特別任命之倫理審查委員會，加以考查、評判及指導，如果適當，才予以核准。此倫理審查委員會，必須獨立於研究者、資助者、或任何其他不當影響力之外。此獨立委員會應遵守該研究實驗所在國的法律及規定。委員會應有權監測進行中的試驗。研究人員有責任向委員會提供實驗監測資訊，特別是任何嚴重不良事件。研究人員應向委員會提供資訊以供審查，包括其研究經費、試驗委託者、所屬機構，及其潛在的利益衝突，和受試驗者參與實驗之誘因。
14. 試驗計畫書需檢附相關倫理考量的聲明，並得符合本宣言所揭櫫之原則。
15. 凡涉及人體試驗的醫學研究，皆須由受過科學訓練的合格人員執行，並由合格臨床醫療人員的監督下進行。對於人體試驗所產生的責任歸屬，皆由合格的醫療人員負責；即使事前已徵得該受試驗者之同意，該受試驗者亦不需負任何責任。
16. 任何有關人體試驗的醫療研究計劃，事前須審慎評估可能的風險、責任、以及對受試驗者或其他人的可能益處。此種評估亦應涵括參與研究的健康志願者。所有研究的設計皆應開放供大眾取得。
17. 除非醫師已充份評估可能產生的風險，並自信能充分地掌控實驗，否則應避免從事有關人體試驗的研究計劃。一旦發現實驗的風險高過其潛在的利益；或已可得到正面或有益之結論時，醫師即應停止其研究計劃。
18. 唯有在研究目的之重要性大於受試驗者可能身受的風險時，有關人體試驗的醫學研究才可以進行。當該受試驗者為健康的志願者時，尤需重視此原則。
19. 唯有被研究的族群可能從醫學研究成果中獲益時，此醫學研究才有其執行之價值。

20. 受試驗者必須是志願參加，並充份瞭解研究內容，才得以參與該項研究計劃。
21. 受試驗者保護其本人身心健全與完整性的權利必須加以尊重。研究人員應採取一切之預防措施，尊重受試驗者之個人隱私，維護其個人資料的私密，並將此研究對其身心健全及人格造成之傷害降到最低。
22. 在任何人體試驗中，每一個可能的受試驗者，必須被告知該研究的目的、方法、經費來源、任何可能的利益衝突、研究人員所屬機構、該研究可預見的益處，及可能伴隨的危險與不適。受試驗者也應被告知其擁有的權利，包括可拒絕參與研究，或可隨時撤回同意而不受報復。在確知受試驗者已充分瞭解以上訊息後，醫師應取得受試驗者於自由意志下簽署之受試同意書，此受試同意書以書面行之為佳。若受試同意書無法以書面方式行之，則非書面之同意必須經過正式地紀錄與見證。
23. 醫師在取得受試同意書時，應特別注意受試驗者是否對醫師有依賴關係，或受試驗者是否在脅迫下行使同意。在此情況下，此受試同意書應由一位充分瞭解全盤研究，但沒有參與研究，並完全與彼無關係的醫師取得。
24. 若受試驗者無法律上之行為能力，或生理或心智上無同意能力，或無法律上行為能力之未成年者，研究人員必須取得符合適用法令之法定代理人受試同意書。除非研究本身有其促進上述族群健康之必要性，而研究又無法於法律上具行為能力之人員上施行，否則此研究不應包涵此類族群。
25. 當一個被視為無法律行為能力之受試驗者，例如未成年之孩童，對參與研究的決定有表達同意之能力時，研究人員除了應取得該受試驗者之同意外，亦必須取得其法定代理人之同意。
26. 當無法從個人取得同意，包括代理人同意或預先同意時，此項對於個人之研究不應進行；除非阻止其簽署受試同意書的個人特殊身心狀況，正是此受試驗者族群的必然特徵。對於此種在無法簽署受試同意書之受試驗者上的研究，研究人員應於試驗計畫書中，陳述其研究之具體原因，以供審查委員會之考量而核准。試驗計畫書中應表明，會儘速從本人，或合法授權之代理人處，取得繼續參與此研究之同意。
27. 作者及出版者皆負道德責任。研究人員在發表研究成果時，即有責任保持其結果的正確性。正面與負面的研究結果都應發表，或可公開取得。研究人員之經費來源，其所屬組織，或研究中任何可能之利益爭議皆應公佈於出版之中。凡不合乎此宣言之原則的實驗報告，皆不該被接受發表。

丙.兼顧醫療照護的醫學研究之附加原則

28. 醫師可以結合醫學研究與醫療照護，但此情況僅止於此研究有潛在的預防、診斷或治療的價值。當醫學研究結合醫療照護時，另有額外的準則來保護這些同為病患和研究對象的人。
29. 一個新醫療方法的益處、危險性、責任、及其效果，應與目前已知最佳的預防、診斷與治療方法對照檢驗。而對於尚無有效預防、診斷與治療方式之研究，不排除使用安慰劑或不予治療來檢驗其療效。
30. 研究結束後，每一個參與研究的病患，都應得到保證其可以接受經此研究證實為最佳的預防、診斷和治療的方法。
31. 醫師應全盤告知病患，那些方面的醫療照護與研究有關。病患的拒絕參與研究，絕對不應影響醫病關係。
32. 在治療病患的過程中，若無有效的預防，診斷和治療的方法，醫師在取得病患之受試同意書後，得以自由採用其判斷下有希望挽救生命，重建健康或減輕痛苦的任何未經證實或新的預防，診斷及治療方法。這些方法，在可能的情況下，應被當作研究的目標，來評估其安全性及有效性。在各種情況下，應將新的消息資訊紀錄，適當時並發表，並應遵守此份宣言的其他相關準則。

附錄二、嚴重不良事件(SAE)/安全性報告作業程序

嚴重不良事件(SAE)/安全性報告作業程序

SOP for SAE/Safety Report

1. 目的

此標準作業程序目的為規範任何經本會(包含臺北醫學大學暨附屬醫院聯合人體研究倫理委員會、臺北醫學大學暨附屬醫院聯合人體研究倫理委員會A、臺北醫學大學暨附屬醫院聯合人體研究倫理委員會B、臺北醫學大學暨附屬醫院聯合人體研究倫理委員會C)核准的試驗/研究進行時所發生的嚴重不良事件（Serious Adverse Event,SAE），及非預期不良事件於追蹤報告時之指引。計畫主持人或委託者於獲知死亡或危及生命之嚴重不良事件，應於獲知日起7 日內通報主管機關及其委託機構，並在獲知日起15 日內提供詳細書面資料。相關規定需依照衛生署所公告之藥品優良臨床試驗準則第106 條規定。

2. 適用範圍

此標準作業程序適用於由計畫主持人、資料與安全監測者、委託者、本會委員或其他相關團體所提報SAE及非預期事件報告之檢視評估。

3. 適用法規及準則

3.1. 赫爾辛基宣言(Declaration of Helsinki) 2008 年中文版，2008
3.2. 人體研究倫理審查委員會組織及運作管理辦法(衛署醫字第 1010265129 號令)，17 August, 2012
3.3. 藥品臨床試驗計畫書主要審核事項(衛署藥字第 0930302777 號公告)，18 February, 2004
3.4. 藥品優良臨床試驗準則(衛署藥字第 0930338510 號公告)，5 January, 20005
3.5. WHO Operational Guidelines for Ethics Committees that Review Biomedical Research，WHO 2000
3.6. Forum for Ethical Review Committees in Asia and the Western Pacific，August 2003
3.7. 倫理審查委員會得簡易程序審查之人體研究案件範圍(衛署醫字第 1010265098C 號)，5 July, 2012
3.8. 醫療法，20 May, 2009
3.9. 人體試驗管理辦法(衛署醫字第 0980263557 號公告)，14 December, 2009
3.10. 人體研究法(總統華總一義字第 10000291401 號令)，28 December, 2011
3.11. 得免取得研究對象同意之人體研究案件範圍(衛署醫字第 1010265083C 號)，5 July, 2012
3.12. 得免倫理審查委員會審查之人體研究案件範圍(衛署醫字第 1010265079 號)，5 July, 2012

4. 職責

在執行過程中，落實嚴重不良事件通報制度，確保試驗/研究執行之品質，以及時偵測試驗/研究藥品或程序可能對受試(訪、檢)者發生傷害，確保受試(訪、檢)者之安全及權益。

5. 定義

5.1. 嚴重不良反應：

依據衛生署公佈之藥品優良臨床試驗準則，試驗期間發生下列嚴重不良反應者，計畫主持人及廠商應呈報政府相關主管單位。嚴重不良反應(SAE/SUSAR)包含以下：
死亡及危及生命
造成永久性殘疾
胎嬰兒先天性畸形
致病人住院或延長病人住院時間
其他可能導致永久性傷害需做處置者
醫療器材之臨床試驗遵照衛生署主管機關所公告之藥品優良臨床試驗準則辦理通報原則。
其他非屬人體試驗或臨床試驗之人體研究，其不同類型之嚴重不良反應或安全性通報亦可

適用本程序

6. 程序概述

步驟	程序	責任歸屬
1	受理「嚴重不良反應事件」通報文件(Registering SAE documents)	行政單位<br>通報者(主持人/臨床護士/臨床研究專員)
2	審查前置作業(Preparing SAE-related documents)	行政單位
3	審查SAE(Reviewing SAE-related documents)	行政單位<br>審查委員
4	文件歸檔儲存(Archiving SAE-related documents)	行政單位

7. 程序

7.1. 受理「嚴重不良反應事件」通報文件(Registering SAE documents)繳交資料：

7.1.1 本會核准之試驗計畫於本體系發生之嚴重不良反應(SAE/ SUSAR)，應依衛生福利部相關法規通報予主管機關及/或本會，本會將進行後續審查程序

7.1.1.1 計畫主持人需在一定時限內通報贊助者，必要時通報主管機關及/或本會

7.1.1.1.1 計畫主持人需向贊助者通報所有嚴重不良事件(SAE)，除非計畫書或其他文件(例如，主持人手冊)定義為不需立即通報者。計畫主持人需依相關法規要求向本會進行SUSAR 通報

非預期的內部不良事件，且涉及新的或增加研究相關風險，應依衛生福利部藥品優良臨床試驗準則第106 條規定，向監管機構及/或本會通報根據衛生福利部藥品優良臨床試驗準則第106 條規定，計畫主持人應立即向贊助者報告任何嚴重不良事件(SAE)，並儘速提供書面報告。非預期嚴重藥品不良反應(SUSAR)，除計畫書或相關文件事前提到者，計畫主持人應先向IRB 報告。即使藥品優良臨床試驗準則第106 條未提到計畫主持人向IRB 報告SUSAR 的時間點，本會參照贊助者向主管機關通報的時間點規定，死亡或危及生命的非預期嚴重藥品不良反應(SUSAR)，計畫主持人應在獲知日起7 日內向本會報告，15 日內提出書面報告。非死亡或危及生命的非預期嚴重藥品不良反應(SUSAR)，應在獲知日起15 日內通報和繳交書面報告給本會。

死亡或危及生命的非預期嚴重藥品不良反應(SUSAR)，贊助者應從贊助者獲知日起7 日內向主管機關報告，並在15 日內提供書面報告。

非死亡或危及生命的非預期嚴重藥品不良反應(SUSAR)，贊助者應於獲知日起15 日之內向主管機關通報並提供書面報告。

不論口頭及/或書面報告，應以受試者代碼代表受試者之身分，不得顯示受試者之姓名、身分證統一編號、住址或其他可辨認受試者身分之資訊。嚴重不良事件與嚴重藥品不良反應之項目由主管機關公告

7.1.1.1.2 非預期的外部不良事件涉及受試(訪、檢)者或其他的風險，發生地研究人員應向贊助者及/或主管機關報告，可提供相關資料予本會，若研究需修正，請依修正案標準作業程序辦理。

7.1.1.1.3 為避免傷害受試(訪、檢)者，未經IRB 事前核准之修正，應於妥善處理後15 日內向本會提出報告。

7.1.1.1.4 若有其他與研究相關，未預期且可能增加受試(訪、檢)者風險的訊息，主持人應於15 日內向贊助者及/或本會報告，以決定是否需要採取進一步行動。

7.1.1.1.5 新資訊顯示可能會對受試(訪、檢)者安全或臨床試驗的進行有不利影響

者，計畫主持人應於獲知日起15 日內向本會報告，並暫停試驗，等待進一步的行動，以保護受試(訪、檢)者。

7.1.1.1.6 任何影響臨床試驗進行或增加受試(訪、檢)者風險事件，計畫主持人應於獲知日起15 日內通報本會，並暫停試驗，等待進一步的行動，以保護受試(訪、檢)者。如果本會終止或暫停臨床試驗時，研究人員應即時通知贊助者。

若本會中止(暫停)或終止已核准之研究，計畫主持人應通知贊助者。若本會中止(暫停)或終止者，屬醫療法第8 條所定之人體試驗，本會需公\文函知主管機構(大多為衛生福利部)

7.1.1.1.7 計畫主持人若為研究藥物許可證持有人，應於5 年藥品監測期前2 年內以每6 個月的頻率，以及第3-5 年每1 年的頻率，向衛生福利部或其他主管機關提交死亡報告或其他特殊不良事件報告。

7.1.1.1.8 若計畫主持人於未事先取得贊助者同意情況下中止(暫停)或終止臨床試驗，計畫主持人應於決定後15 日內通知試驗機構、贊助者及本會。

7.1.1.1.9 關於通報死亡，計畫主持人需提供贊助者及本會任何額外要求的資料(如解剖報告和醫療報告)

7.1.1.1.10計畫主持人應依計畫書定義之報告範圍及時程，向贊助者通報可能影響試驗安全評估的不良事件或實驗室異常值。

7.1.1.1.11臨床試驗結束後，計畫主持人應通知機構及本會有關試驗的結果摘要，並提供主管機關所需的任何報告

7.1.2 通報者須填寫下列文件並依序檢附，以進行「嚴重不良反應初始報告」；並於受試(訪、檢)者住院期間**每10 天**繳交一次**追蹤報告**，直至症狀解除或出院時再次繳交追蹤報告。依序檢附為：

1. 嚴重不良事件/安全性報告通報回函
2. 送審資料清單
3. 計畫書摘要
4. 臨床試驗嚴重不良反應 - 主持人評估表(安全性報告可免附此份評估表)
5. 衛生署藥物不良反應通報表-藥物
6. 衛生署藥物不良反應通報表-醫材
7. 臨床/研究 SAE 審查意見表(藥物)
8. 臨床/研究 SAE 審查意見表(非藥物)
9. 試驗/研究最近一次核准函
10. 相關附件資料(如：病歷摘要及相關檢查資料等)

7.1.3 行政單位需核對上述送審資料之完整性，若資料不齊全應盡速通知通報者補齊完整資料，以進行後續審查程序

7.1.4 本會核准之試驗計畫中，與試驗主題(藥品、醫材等)相關，或該試驗計畫於國外或國內其他醫院所發生之非預期嚴重不良反應，均應通報法規機關及本會，由行政單位依據安全性報告方式，檢附7.1.2 資料進行評估報備，將文件歸檔儲存後續備查

7.2. 審查前置作業(Preparing SAE-related documents)

7.2.1 行政單位確認「嚴重不良反應事件」通報文件之完整性後，將SAE 案件於5 個工作天內送委員評估。「安全性報告」於每週/每月送委員評估存查

7.2.2 藥品類「嚴重不良反應事件」由本體系試驗機構之本會藥事背景委員依據**TMU-JIRB Form065 試驗/研究SAE 審查意見表(藥物)**進行評估審查

7.2.3 非藥品類「嚴重不良反應事件」由總召集人指定1 位委員依據 **TMU-JIRBForm086 試驗/研究SAE 審查意見表（非藥物類）**進行評估

7.2.4 行政單位應將案件相關資料同時紀錄至資料庫備查

7.3. 審查SAE(Reviewing SAE-related documents)

執行程序:

7.3.1 死亡案例之嚴重不良反應事件評估

行政單位收到通報後5 個工作天內交由相關評估人員，於5 個工作天內完成評估。

7.3.2 非死亡案例之嚴重不良反應事件評估

7.3.2.1 行政單位收到通報後5 個工作天內交由相關評估人員，於5 個工作天內完成評估

7.3.2.2 行政單位人員取得評估報告後，連同完整「嚴重不良反應事件」通報文件呈送執行秘書及總召集人簽核。當評估結果為「送給醫療委員做進一步審查」時，由總召集人決定增加1 至2 位醫療專家審查；當評估結果為「存查」，將文件留存備查並於最近期會議中報告；若討論時因該SAE 報告而對其安全性有疑慮時，由主任委員/主席決定是否需增加1 至2 位專家審查

7.3.2.3 行政單位將完整「嚴重不良反應事件通報文件」、**TMU-JIRB Form 065 試驗/研究SAE 審查意見表(藥物)或TMU-JIRB Form086 試驗/研究SAE 審查意見表（非藥物類）**呈交總召集人/主任委員指派之專家進行審查。專家審查結果紀錄於 **TMU-JIRB Form039 試驗/研究SAE 審查通知表**，經執行秘書及總召集人確核過後，由行政單位依下述流程處理後續程序，並得通知主持人決議

7.3.3 審查意見：

專家針對該SAE 之審查意見為「無須修改」或「建議修改」，若建議召開臨時會、中止(暫停)/終止該計畫，則由行政單位報告總召集人後依各該作業程序進行

7.3.3.1 「無須修改」：行政單位需將該SAE 於會中報備存查

7.3.3.2 「建議修改」：專家需填寫建議修改原因，並選擇建議修改之試驗/研究文件

7.3.4 審查結果：專家應依「審查意見」做出審查建議為「存查」、「修正相關文件」、「暫停該試驗/研究納入新受試(訪、檢)者」、「提會討論」、「中止(暫停)/終止該計畫」、「其他」

7.3.4.1 「存查」：行政單位將所有SAE 通報文件及相關評估審查建議於會議中報告，並留存備查

7.3.4.2 「提會討論」：當審查建議為「請主持人針對審查意見提出說明」、「建議修改試驗/研究計畫書」、「建議修改受試(訪、檢)者同意書」時，由行政單位通知試驗/研究主持人/贊助廠商決議，並提供 **TMU-JIRB Form087 試驗/研究主持人回覆SAE 審查結果說明表**文件，請主持人針對審查意見提出說明。主持人完成後交回本會，由行政單位送交會議討論並遵循決議

7.3.4.3 「暫停該試驗/研究納入新受試(訪、檢)者」：當嚴重不良反應報告所提供之資料經醫療專家判斷恐對受試(訪、檢)者之權益產生疑慮時，得依其專業能力，提出建議「暫停該試驗/研究納入新受試(訪、檢)者」。惟此項建議需由主任委員及總召集人同意後，始得以本會名義通知該主持人及贊助廠商。主任委員及總召集人於同意「暫停該試驗/研究納入新受試(訪、檢)者」時，應由行政單位於10 個工作天內召開臨時會，若該計畫原審查委員會之例行審查會議於此時限內，得直接排入臨時動議討論，不另行召開臨時會

7.3.4.4 「中止(暫停)/終止該計畫」：當嚴重不良反應報告所提供之資料經醫療專家判斷恐對受試(訪、檢)者之權益產生疑慮時，得依其專業能力，提出建議「中止(暫停)/終止該計畫」。惟此項建議需由總召集人同意後，依本會相關作業程序進行

7.3.4.5 「其他」：專家得依其專業能力作出「其他」審查結果建議，提報委員會進行決議

7.3.4.6 如SAE 審查結果需召開臨時會，其程序參照**TMU-JIRB SOP022 議程製作、會**

議程序與會議紀錄作業程序。

7.4. 會議報備程序(Reporting SAE-related documents to board meeting)

7.4.1 行政單位需將經相關評估人員評估之SAE 案件(初始報告/追蹤報告出現受試(訪、檢)者情況惡化)，於最近之該計畫原審查委員會審查會議報備該SAE 案件及其相關評估或審查結果，並提供一式兩份相關文件供與會委員翻閱

7.4.2 當嚴重不良反應之審查結果經主任委員及總召集人同意以本會名義發出「暫停該試驗/研究納入新受試(訪、檢)者」之要求時，該嚴重不良反應報告應於臨時會進行討論或於該計畫原審查委員會例行審查會議中，依臨時動議項目進行討論

7.4.3 行政單位應依會議討論之結果，於10 個工作天內，進行該計畫之後續溝通及處理

7.5. 文件歸檔儲存(Archiving SAE-related documents)

7.5.1 行政單位需持續收集該SAE 案例之追蹤報告，連同相關審查記錄文件一併存檔備查

7.5.2 如SAE 之追蹤報告出現受試(訪、檢)者情況惡化之報告，需提送主任委員，並自 7.3 審查SAE 程序之流程，再次由本會評估人員確認SAE 之嚴重程度，及相關之醫療專家審查程序，並依結果通知主持人或贊助廠商

7.5.3 SAE 相關文件直接歸檔入該試驗/研究檔案進行存查

8. 附件

附件一TMU-JIRB Form016 送審資料清單-SAE
附件二TMU-JIRB Form038 試驗/研究SAE 通報回函
附件三TMU-JIRB Form039 試驗/研究SAE 審查通知表
附件四TMU-JIRB Form065 試驗/研究SAE 審查意見表(藥物)
附件五TMU-JIRB Form086 試驗/研究SAE 審查意見表（非藥物類）
附件六TMU-JIRB Form087 試驗/研究主持人回覆SAE 審查結果說明表
附件七TMU-JIRB SOP022 議程製作、會議程序與會議記錄作業程序
附件八TMU-JIRB Form097 衛生署藥物不良反應通報表-醫材
附件九TMU-JIRB Form098 衛生署藥物不良反應通報表-藥物
附件十TMU-JIRB Form080 試驗/研究SAE-主持人評估表
