# Supplementary material for: Assessment of the clinical efficacy of the heart spectrum blood pressure monitor for diagnosis of atrial fibrillation: An unblinded clinical trial
Source: PLoS One. 2018 Jun 14;13(6):e0198852. doi: 10.1371/journal.pone.0198852 (PMC6001975; doi:10.1371/journal.pone.0198852)
Supplement: S2 File — (DOC) [file pone.0198852.s002.doc]

**Trial Title: Assessment of the Clinical Efficacy of the Heart Spectrum Blood Pressure Monitor for Diagnosis of Atrial Fibrillation: An Unblinded Clinical Trial**

**Ethics Ref:** TMU-Joint Institutional Review Board/ TMU JIRB No.: N201510061

**EudraCT Number**: ClinicalTrials.gov ID: NCT03095131

**Date and Version No**: 20150505/version1.0

| **Chief Investigator:** | Wei-Fong Kao, MD. PhD., Department of Emergency Medicine, School of Medicine, College of Medicine, Taipei Medical University, Taipei, Taiwan, wfkao100a@hotmail.com.tw |
| --- | --- |
| **Investigators:** | Sen-Kuang Hou, MD. PhD., Department of Emergency Medicine, Taipei Medical University Hospital, Taipei, Taiwan, 992001@h.tmu.edu.tw  Chun-Yao Huang, MD. PhD., Division of Cardiology and Cardiovascular Research Center, Department of Internal Medicine, Taipei Medical University Hospital, Taipei, Taiwan, cyhuang@tmu.edu.tw  Chun-chieh Chao MD., MS., Department of Internal Medicine, School of Medicine, College of Medicine, Taipei Medical University, Taipei, Taiwan, chaosees@gmail.com  Chung-Chih Cheng, PhD., Department of Medical Device, Medical and Pharmaceutical Industry Technology and Development Center, Taipei, Taiwan, allencheng@pitdc.org.tw  Yi-Jung Chen, MS., Department of Medical Device, Medical and Pharmaceutical Industry Technology and Development Center, Taipei, Taiwan, yirong1976@pitdc.org.tw |
| **Sponsor:** | OSTAR MEDITECH CORP.  (5F., 46-4 Min-Chiuan Rd., Shing-Tien Dist., New Taipei City 231, Taiwan) |
| **Funder:** | Medical and Pharmaceutical Industry Technology and Development Center |
| **Chief Investigator Signature:**  **Statistician Signature:** | 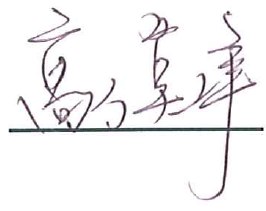  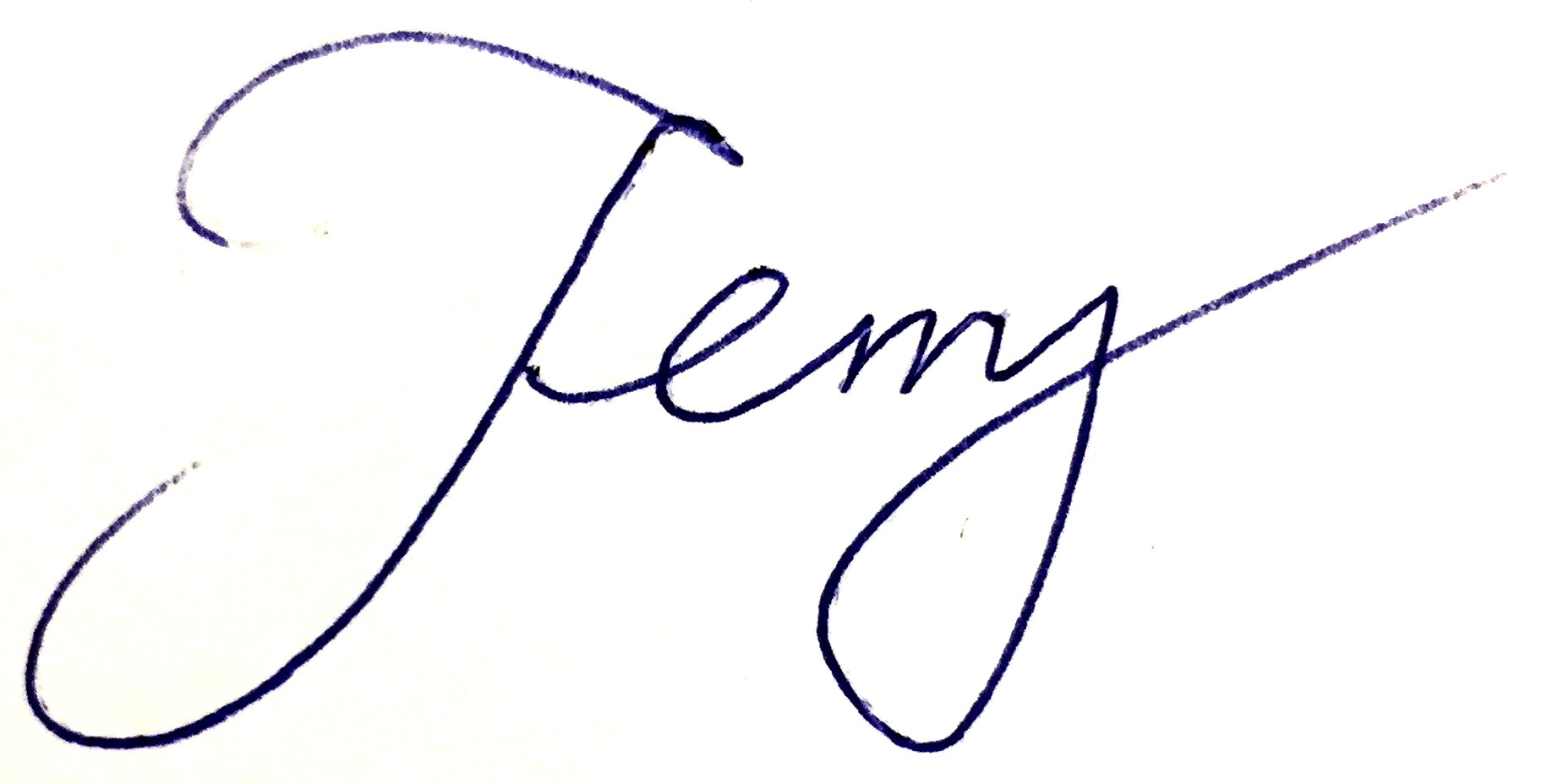 |

This manuscript has not been published or presented elsewhere in part or in entirety and is not under consideration by another journal. All the authors have approved the manuscript and agree with submission to your esteemed journal. There are no conflicts of interest to declare. All study participants provided informed consent, and the study design was approved by the appropriate ethics review board. We have read and understood your journal’s policies, and we believe that neither the manuscript nor the study violates any of these. There are no conflicts of interest to declare.

**Confidentiality Statement**

This document contains confidential information that must not be disclosed to anyone other than the Sponsor, the Investigator Team, HRA, host organisation, and members of the Research Ethics Committee, unless authorised to do so.

# KEY TRIAL CONTACTS

| **Chief Investigator** | Wei-Fong Kao, MD. PhD., Taipei Medical University Hospital  Email: wfkao100a@hotmail.com.tw  Phone: 886-2-27372181 #8107 |
| --- | --- |
| **Sponsor** | OSTAR MEDITECH CORP.  Email: ostar@ostar.com.tw  Phone: 886-2-29182390  Fax: 886-2-29182683 |
| **Clinical Trials Unit** | Taipei Medical University Hospital  Email: ostar@ostar.com.tw  Phone: 886-2-27372181 |
| **Statistician** | Yung-Cheng Wu, OSTAR MEDITECH CORP.  Email: terry@ostar.com.tw  Phone: 886-2-29182390  Fax: 886-2-29182683 |
| **Committees** | TMU-Joint Institutional Review Board  Email: ostar@ostar.com.tw  Phone: 886-2-27372181 |

# SYNOPSIS

| Trial Title | Assessment of the Clinical Efficacy of the Heart Spectrum Blood Pressure Monitor for Diagnosis of Atrial Fibrillation: An Unblinded Clinical Trial | |
| --- | --- | --- |
| Internal ref. no. (or short title) | 23 | |
| Clinical Phase | N/A | |
| Trial Design | The physician used a 12-lead ECG and the Heart Spectrum Blood Pressure Monitor simultaneously. The Heart Spectrum Blood Pressure Monitor used an FFT analysis for the diagnosis of AF; the findings were compared with the readings from the 12-lead ECG. | |
| Trial Participants | Twenty-nine subjects with AF and 33 without AF, aged 25 to 97 years (mean: 63.5 years), were included.  Intervention(s) for clinical trials Subjects who had been exposed to high frequency surgical equipment during testing, those with cardiac pacemakers or implantable defibrillators, and pregnant women were excluded. | |
| Planned Sample Size | 62 | |
| Treatment duration | February 22, 2016 to June 23, 2016 | |
| Follow up duration | February 22, 2016 to December 7, 2016 | |
| Planned Trial Period | N/A | |
|  | Objectives | Outcome Measures |
| Primary | To compare the diagnostic efficacy of the Heart Spectrum Blood Pressure Monitor with a 12-lead ECG in patients with AF. Three measurement methods were used in this study: blood pressure, mean arterial pressure (MAP) which is calculated from individual blood pressure as constant pressure, and a constant pressure 60 mmHg, in order to analyze the heart index and compare it with simultaneous 12-lead ECG. | The sensitivity, specificity, positive predictive value (PPV), and negative predictive value (NPV). |
| Secondary | N/A | N/A |
| Investigational Medicinal Product(s) | N/A | |
| Formulation, Dose, Route of Administration | N/A | |

# ABBREVIATIONS

| AF | Atrial Fibrillation |
| --- | --- |
| AFL | Atrial Flutter |
| CFR | Case Report Form |
| ECG | Electrocardiography |
| EPS | Electrophysiologic study |
| FFT | Fast Fourier Transformation |
| GCP | Good Clinical Practice |
| ICF | Informed Consent Form |
| ITT | Intent to Treat |
| MVR | Moderate Ventricular Response |
| PP | Per protocol |
| PAC | Premature Atrial Contraction |
| PVC | Premature Ventricular Contraction |
| PSVT | Paroxysmal Supraventricular Tachycardia |
| RVR | Rapid Ventricular Response |
| SAE | Serious Adverse Event |
| SVR | Slow Ventricular Response |
| VF | Ventricular Fibrillation |
| VT | Ventricular Tachycardia |

# BACKGROUND AND RATIONALE

Atrial fibrillation (AF) is a common cardiac arrhythmia. There are an estimated 2.2 million people (median age of approximately 75 years) in the United States with AF.^1^ It is worth noting that from 1985 through 1999, hospitalizations increased from 154,086 to 376,487 for a first-listed diagnosis.^2^ Overall, the age-standardized rate (per 100,000) increased from 27.6 in 1980 to 69.8 in 1998 (an average annual increase of 5.4%, p < 0.0001).^3^

In general, the occurrence of AF with atrial degeneration is related. Therefore, the higher the age of the population, the higher the incidence of AF. The estimated prevalence of AF is 0.4% to 1% in the general population and increases with age up to 5% to 6% in the population aged > 65 years, and to 8% among those ≥ 80 years old. Similarly, while the incidence of AF is < 0.1% per year for those aged < 40 years, it increases to above 1.5% per year in women and 2% in men aged > 80 years.^4^

In addition, patients with hypertension, heart failure, valvular heart disease, diabetes, or hyperthyroidism are more prone to have AF.^5,6^

The incidence of stroke is more than 4 times higher in patients with cardiac failure (p-value < 0.001) and 5 times higher in those with AF (p-value <0.001).^7^ Patients with AF require physician treatment to prevent the occurrence of stroke.

With the ageing population, the number of people experiencing AF continues to rise. The age-adjusted incidence of stroke has more than doubled for patients with coronary heart disease, and more than tripled for those with hypertension. In persons with coronary heart disease or cardiac failure, AF doubles the risk of stroke in men and triples it in women.^7-11^

Anticoagulant therapy is effective in reducing the risk of systemic embolization in patients with nonvalvular AF. Anticoagulation with warfarin, dabigatran, rivaroxaban, apixaban, or edoxaban reduces this risk by almost 70%.^12^

Evaluation by 12-lead and dynamic ECG can diagnose most cases of AF.^13-15^ However, 12-lead ECG needs to be performed and interpreted at a medical institution by physicians; this cannot be implemented by the user in the home environment.

The Heart Spectrum Blood Pressure Monitor is a simple, home-use device that can detect the pressure change in the blood vessels of the heart, and the measurement undergoes fast Fourier transformation (FFT) to calculate the heart beat frequency and accurate blood pressure data. It can also analyze the frequency of heart beat to determine if AF has occurred.

# OBJECTIVES AND OUTCOME MEASURES

| **Objectives** | **Outcome Measures** | **Timepoint(s) of evaluation of this outcome measure (if applicable)** |
| --- | --- | --- |
| **Primary Objective** To compare the diagnostic efficacy of the Heart Spectrum Blood Pressure Monitor with a 12-lead ECG in patients with AF. Three measurement methods were used in this study: blood pressure, mean arterial pressure (MAP) which is calculated from individual blood pressure as constant pressure, and a constant pressure 60 mmHg, in order to analyze the heart index and compare it with simultaneous 12-lead ECG. | The sensitivity, specificity, positive predictive value (PPV), and negative predictive value (NPV). | monitoring for about 60 seconds per test |

# TRIAL DESIGN

The physician used a 12-lead ECG and the Heart Spectrum Blood Pressure Monitor simultaneously. The Heart Spectrum Blood Pressure Monitor used an FFT analysis for the diagnosis of AF; the findings were compared with the readings from the 12-lead ECG.

# PARTICIPANT IDENTIFICATION

## Trial Participants

Twenty-nine subjects with AF and 33 without AF, aged 25 to 97 years (mean: 63.5 years), were included.

Intervention(s) for clinical trials Subjects who had been exposed to high frequency surgical equipment during testing, those with cardiac pacemakers or implantable defibrillators, and pregnant women were excluded.

## Inclusion Criteria

- Subjects older than 20 years of age who had been diagnosed with AF using 12-lead ECG, or were healthy (without AF) were included.

## Exclusion Criteria

- Subjects who had been exposed to high frequency surgical equipment during testing, those with cardiac pacemakers or implantable defibrillators, and pregnant women were excluded.

# TRIAL PROCEDURES

The physician applied the 12-lead ECG patches on the subject's body and attached the blood pressure cuff to the subject's arm. Then the Heart Spectrum Blood Pressure Monitor was connected to a computer for collecting data. The 12-lead ECG and Heart Spectrum Blood Pressure Monitor were used simultaneously. The Heart Spectrum Blood Pressure Monitor calculated the heart spectrum results via FFT analysis for the diagnosis of AF. The clinical trial results were judged by the professional physician.

## Screening and Eligibility Assessment

This clinical trial was conducted in an open study (non-blinded). In the emergency room, when medical staff found patients with AF, they informed the research physicians to study the case. The Heart Spectrum Blood Pressure Monitor was compared with 12-lead ECG (ELI 250) after written informed consent was obtained from the patient. The results were interpreted by the emergency physician. Finally, the sensitivity, specificity, positive predictive value (PPV), and negative predictive value (NPV) were analyzed to evaluate the clinical efficacy.

## Informed Consent

The participant must personally sign and date the Informed Consent form before any trial specific procedures are performed.

Written and verbal versions of the Participant Information and Informed Consent will be presented to the participants detailing no less than: the exact nature of the trial; what it will involve for the participant; the implications and constraints of the protocol; the known side effects and any risks involved in taking part. It will be clearly stated that the participant is free to withdraw from the trial at any time for any reason without prejudice to future care, without affecting their legal rights and with no obligation to give the reason for withdrawal.

The participant will be allowed as much time as wished to consider the information, and the opportunity to question the Investigator, their GP or other independent parties to decide whether they will participate in the trial. Written Informed Consent will then be obtained by means of participant dated signature and dated signature of the person who presented and obtained the Informed Consent. The person who obtained the consent must be suitably qualified, and have been authorised to do so by the Chief/Principal Investigator. A copy of the signed Informed Consent will be given to the participant. The original signed form will be retained at the trial site.

## Baseline Assessments

To compare the diagnostic efficacy of the Heart Spectrum Blood Pressure Monitor with a 12-lead ECG in patients with AF. Three measurement methods were used in this study: blood pressure, mean arterial pressure (MAP) which is calculated from individual blood pressure as constant pressure, and a constant pressure 60 mmHg, in order to analyze the heart index and compare it with simultaneous 12-lead ECG.

## Discontinuation/Withdrawal of Participants from Trial Treatment

Each participant has the right to withdraw from the trial at any time. In addition, the Investigator may discontinue a participant from the trial at any time if the Investigator considers it necessary for any reason including:

- Researchers are unable to comply with the clinical trial protocol or the Good Clinical Practice (GCP).
- Security considerations.
- There is sufficient information to confirm the lack of efficacy of test equipment.
- The investigator did not recruit enough subjects.

## Definition of End of Trial

The end of trial is the date of the last visit of the last participant.

# SAFETY REPORTING

## Definitions

| Adverse Event (AE) | Any untoward medical occurrence in a participant to whom a medicinal product has been administered, including occurrences which are not necessarily caused by or related to that product. |
| --- | --- |
| Adverse Reaction (AR) | An untoward and unintended response in a participant to an investigational medicinal product which is related to any dose administered to that participant.  The phrase "response to an investigational medicinal product" means that a causal relationship between a trial device and an AE is at least a reasonable possibility, i.e. the relationship cannot be ruled out.  All cases judged by either the reporting medically qualified professional or the Sponsor as having a reasonable suspected causal relationship to the trial device qualify as adverse reactions. |
| Serious Adverse Event (SAE) | A serious adverse event is any untoward medical occurrence that:   - results in death - is life-threatening - requires inpatient hospitalisation or prolongation of existing hospitalisation - results in persistent or significant disability/incapacity - consists of a congenital anomaly or birth defect.   Other ‘important medical events’ may also be considered serious if they jeopardise the participant or require an intervention to prevent one of the above consequences.  NOTE: The term "life-threatening" in the definition of "serious" refers to an event in which the participant was at risk of death at the time of the event; it does not refer to an event which hypothetically might have caused death if it were more severe. |
| Serious Adverse Reaction (SAR) | An adverse event that is both serious and, in the opinion of the reporting Investigator, believed with reasonable probability to be due to one of the trial treatments, based on the information provided. |
| Suspected Unexpected Serious Adverse Reaction (SUSAR) | A serious adverse reaction, the nature and severity of which is not consistent with the information about the medicinal product in question set out:   - in the case of a product with a marketing authorisation, in the summary of product characteristics (SmPC) for that product - in the case of any other investigational medicinal product, in the investigator’s brochure (IB) relating to the trial in question. |

NB: to avoid confusion or misunderstanding of the difference between the terms “serious” and “severe”, the following note of clarification is provided: “Severe” is often used to describe intensity of a specific event, which may be of relatively minor medical significance. “Seriousness” is the regulatory definition supplied above.

Any pregnancy occurring during the clinical trial and the outcome of the pregnancy should be recorded and followed up for congenital abnormality or birth defect, at which point it would fall within the definition of “serious”.

## Procedures for Recording Adverse Events

All AEs occurring during the trial / or until < Insert appropriate time period during which AEs will be recorded > that are observed by the Investigator or reported by the participant, will be recorded on the CRF.

The following information will be recorded: description, date of onset and end date, severity, assessment of relatedness to trial device, other suspect drug or device and action taken. Follow-up information should be provided as necessary.

The severity of events will be assessed on the following scale: 1 = mild, 2 = moderate, 3 = severe.

AEs considered related to the trial device as judged by a medically qualified investigator or the Sponsor will be followed either until resolution, or the event is considered stable.

It will be left to the Investigator’s clinical judgment to decide whether or not an AE is of sufficient severity to require the participant’s removal from treatment. A participant may also voluntarily withdraw from treatment due to what he or she perceives as an intolerable AE. If either of these occurs, the participant must undergo an end of trial assessment and be given appropriate care under medical supervision until symptoms cease, or the condition becomes stable.

# STATISTICS

## Description of Statistical Methods

At the end of the trial, the heart spectrum blood pressure monitor will be used to differentiate the sensitivity and specificity of the diagnosis of atrial fibrillation.

## The Number of Participants

This clinical study is expected to be performed at the Taipei Medical University Hospital (Single Clinical Trials Center). It is expected that 60-100 subjects will be recruited as a feasibility study to assess the clinical efficacy of the heart spectrum blood pressure monitor.

## Criteria for the Termination of the Trial

Anything that deviates from the original statistical design needs to be justified and must be described and explained in the final report.

## Procedure for Accounting for Missing, Unused, and Spurious Data.

When the test results can not be used as a physician to diagnose the condition of the heart, this data will not be included in the statistical analysis of efficacy evaluation. For unused data, the trial physician must analyze the probable factors that trigger the situation and record it in the case report form.

## Inclusion in Analysis

Subjects older than 20 years of age who had been diagnosed with AF using 12-lead ECG, or were healthy (without AF) were included. And this is an unblinded clinical trial.

## Procedures for Reporting any Deviation(s) from the Original Statistical Plan

Any deviation(s) from the original statistical plan should be described and justified in protocol and/or in the final report, as appropriate.

# DATA MANAGEMENT

## Access to Data

Direct access will be granted to authorised representatives from the Sponsor, host institution and the regulatory authorities to permit trial-related monitoring, audits and inspections.

## Data Recording and Record Keeping

(1) The clinical trial data will be processed by the designated researchers, the computer file will be encrypted, the relevant documents will be stored in the locked cabinet, the key kept by the researchers.

(2) Test moderators and researchers need to systematically focus on the management of clinical research related documents, to facilitate the trial commissioner or other authorized at any time to review. Clinical related documents include:

- The case folder must contain the complete case report form, the subject consent form and other relevant information.
- The study materials shall contain the books and periodicals, all correspondence with the trial commissioner or the Human Test Committee, and other relevant information.
- Use of test equipment and all relevant documents.

(3) Without the consent of the test moderator and the test commissioner, no research document can be destroyed. Researchers or research institutions should take steps to prevent these files or documents from being destroyed in advance.

(4) If the responsible researcher is no longer responsible for the preservation of the study record due to retirement, withdrawal or otherwise, the supervisory task must be delivered to another person who is willing to take the responsibility and notify the trial commissioner in writing the name and contact address of this new person in charge.

(5) The test moderator must adhere to the subject data confidentiality principle, so the case report form or other documents provided to the trial commissioner can only identify the subject by the abbreviation of the name or the subject number. A document that is not provided to the commissioner (for example, a signed consent form) must be strictly managed by the test moderator.

The participants will be identified by a unique trial specific number and/or code in any database. The name and any other identifying detail will NOT be included in any trial data electronic file.

# ETHICAL AND REGULATORY CONSIDERATIONS

This study did not address the ethical concerns of placebo control.

This product has passed the electrical safety test, the safety of the subject has been carefully assessed and considered.

This test will comply with the Helsinki Declaration (Helsinki Declaration), the "medical device"

(GCP) "and" Taipei Medical University and Affiliated Hospital Joint Human Body Research Ethics Committee "standard implementation of clinical trials.

The program by the "Taipei Medical University and Affiliated Hospital Joint Human Body Research Ethics Committee" after the audit can be implemented. If necessary, the test moderator to the "Taipei Medical University Joint Human Body Research Ethics Committee" report the progress of the implementation of the trial.

The trial moderator should inform the subject in detail of the purpose and method of the trial, the expected effect, the possibility of side effects and the risk, and obtain the consent of the recipient.

The design of this test is based on the protection of the rights of the subject, such as when the test equipment is hazardous to the health of the subject when disposed of in other treatments.

# FINANCE AND INSURANCE

All expenses incurred in this trial are provided by the test commissioner and the subject is not required to pay any money.

The commissioning officer shall bear the medical injury liability arising from the test equipment, but shall not be liable for medical injury resulting from improper or neglected medical treatment.

If the clinical trial program implementation of adverse reactions or injury, the trial commissioner will provide the subject of professional medical care and medical advice. However, the adverse effects reported in this project, or the expected damage caused by these adverse reactions, the trial commissioner will not be compensated or compensated.

# REFERENCES

[1] Feinberg WM, Blackshear JL, Laupacis A, Kronmal R, Hart RG. Prevalence, age distribution, and gender of patients with Af. Analysis and implications. *Arch Intern Med.* 1995;155(5):469-473.

[2] Wattigney WA, Mensah GA, Croft JB. Increasing trends in hospitalization for Af in the United States, 1985 through 1999: Implications for primary prevention. *Circulation.* 2003;108:711-716.

[3] Wattigney WA, Mensah GA, Croft JB. Increased Af mortality: United States, 1980–1998. *Am J Epidemiol.* 2002; 155: 819-826.

[4] Pérula-de-Torres LA, Martínez-Adell MA, González-Blanco M, et al. Collaborative Group DOFA-AP. Opportunistic detection of AF in subjects aged 65 years or older in primary care: a randomised clinical trial of efficacy. DOFA-AP study protocol. *BMC Fam Pract.* 2012;13:13-106.

[5] Stewart S, Hart CL, Hole DJ, McMurray JJ. A population-based study of the long-term risks associated with Af: 20- year follow-up of the Renfrew/Paisley study. *Am J Med.* 2002;113:359-364.

[6] Benjamin EJ, Levy D, Vaziri SM, D’Agostino RB, Belanger AJ, Wolf PA. Independent risk factors for Af in a population-based cohort. The Framingham Heart Study. *JAMA.* 1994;271:840-844.

[7] Wolf PA, Abbott RD, Kannel WB. Af as an independent risk factor for stroke: the Framingham Study. *Stroke.* 1991; 22(8):983-988.

[8] Benjamin EJ, Wolf PA, D'Agostino RB, Silbershatz H, Kannel WB, Levy D. Impact of Af on the risk of death: the Framingham Heart Study. *Circulation.* 1998;98(10):946-952.

[9] Conen D, Chae CU, Glynn RJ, et al. Risk of death and cardiovascular events in initially healthy women with new-onset Af. *JAMA.* May 25, 2011;305(20):2080-2087.

[10] Wang TJ, Larson MG, Levy D, et al. Temporal relations of Af and congestive heart failure and their joint influence on mortality: the Framingham Heart Study. *Circulation.* 2003;107(23):2920-2925.

[11] Conen D, Osswald S, Albert CM. Epidemiology of Af. *Swiss Med Wkly.* 2009;139(25–26):346-352.

[12] Warren JM, Daniel ES, Gregory YHL, et al. Af: Anticoagulant therapy to prevent embolization. ©UpToDate. August, 2016.

[13] Fung E, Järvelin MR, Doshi RN, et al. Electrocardiographic patch devices and contemporary wireless cardiac monitoring. *Front Physiol.* 2015;6:149.

[14] Camm AJ, Lip GY, De Caterina R, et al; ESC Committee for Practice Guidelines (CPG). 2012 focused update of the ESC Guidelines for the management of Af: an update of the 2010 ESC Guidelines for the management of Af. Developed with the special contribution of the European Heart Rhythm Association. *Eur Heart J.* 2012;33(21):2719-2747.

[15] Rosero SZ, Kutyifa V, Olshansky B, Zareba W. Ambulatory ECG monitoring in Af management. *Prog Cardiovasc Dis.* 2013;56(2):143-152.

[16] Barrett PM, Komatireddy R, Haaser S, et al. Comparison of 24-hour Holter monitoring with 14-day novel adhesive patch electrocardiographic monitoring. *Am J Med.* 2014;127(1):95.e11-17.

[17]  Joseph W. Home Monitoring for Af Using a Microlife Blood Pressure Monitor (TRIPPS.) *ClinicalTrials.gov Identifier: NCT00861354.* March 10, 2009.

[18] Riyaz AK. Six Lead Identification of Af (SL-AF.) *ClinicalTrials.gov Identifier: NCT02124629.* March 24, 2015.

[19] Ajijola OA, Boyle NG, Shivkumar K. Detecting and monitoring arrhythmia recurrence following catheter ablation of Af. *Front Physiol.* 2015; 6:90.

[20] Barrett PM, Komatireddy R, Haaser S, et al. Comparison of 24-hour Holter monitoring with 14-day novel adhesive patch electrocardiographic monitoring. *Am J Med.* 2014;127(1):95.e11-17.

[21] Higgins P, MacFarlane PW, Dawson J, McInnes GT, Langhorne P, Lees KR. Noninvasive cardiac event monitoring to detect Af after ischemic stroke: a randomized, controlled trial. *Stroke.* 2013;44(9):2525- 2531.

[22] Vaes B, Stalpaert S, Tavernier K, et al. The diagnostic accuracy of the MyDiagnostick to detect Af in primary care. *BMC Fam Pract.* 2014;15:113.

[23] Tieleman RG, Plantinga Y, Rinkes D, et al. Validation and clinical use of a novel diagnostic device for screening of Af. *Europace.* 2014;16(9):1291-1295.
