# Supplementary material for: Assessment of the clinical efficacy of the heart spectrum blood pressure monitor for diagnosis of atrial fibrillation: An unblinded clinical trial
Source: PLoS One. 2018 Jun 14;13(6):e0198852. doi: 10.1371/journal.pone.0198852 (PMC6001975; doi:10.1371/journal.pone.0198852)
Supplement: S3 File — (DOC) [file pone.0198852.s003.doc]

**STATISTICAL ANALYSIS PLAN**

**Statistical Analysis Plan (SAP)**

**Version**: <1.0>

**Author**: <Terry Wu, OSTAR MEDITECH CORP.>

**Date**: 23-03-2017

**CONTENTS**

1 INTRODUCTION

Atrial fibrillation (AF) is the most common type of cardiac arrhythmia. Patients with asymptomatic and undiagnosed AF are at increased risk of heart failure and ischaemic stroke. It is, therefore, important to be able to identify this condition as early as possible. A 12-lead electrocardiogram (ECG) is the gold-standard for diagnosing AF, however, it is not the most convenient process for patients or technicians. ‘Blood Pressure and Heart Spectrum Monitor’ is a non-invasive digital blood pressure device using oscillometric technique to measure blood pressure, heartbeat frequency, and heart noise. It is intended to discriminate AF from a normal cardiac rhythm and can be used as a home-monitoring tool for management of cardiovascular disease. In order to evaluate the clinical effectiveness of ‘ Blood Pressure and Heart Spectrum Monitor’ for the detection of AF, a study was designed to explore its diagnostic accuracy. Comparative study of the device with the gold-standard diagnostic method (ECG) will be undertaken in 60 to 100 subjects. Sensitivity and specificity will be determined to demonstrate the clinical performance of ‘Blood Pressure and Heart Spectrum Monitor’.

2 DATA SOURCE

2.1 Subjects older than 20 years of age who had been diagnosed with AF using 12-lead ECG, or were healthy (without AF) were included.

2.2 Subjects who had been exposed to high frequency surgical equipment during testing, those with cardiac pacemakers or implantable defibrillators, and pregnant women were excluded.

3 ANALYSIS OBJECTIVES

The Heart Spectrum Blood Pressure Monitor can be used at home, and is capable of determining the occurrence of AF. Three measurement methods were used in this study. The sensitivity, specificity, PPV, and NPV of the methods used are between 90% and 100%, and the Heart Spectrum Blood Pressure Monitor can be effectively applied to AF detection.

The Heart Spectrum Blood Pressure Monitor can be used at home and can replace the need for an in-office ECG, thereby accomplishing meaningful telecare.

To compare the diagnostic efficacy of the Heart Spectrum Blood Pressure Monitor with a 12-lead ECG in patients with AF. Three measurement methods were used in this study: blood pressure, mean arterial pressure (MAP) which is calculated from individual blood pressure as constant pressure, and a constant pressure 60 mmHg, in order to analyze the heart index and compare it with simultaneous 12-lead ECG.

(1) Method 1: Standard blood pressure measurement was used to determine the heart index and compare with 12-lead ECG synchronously.

(2) Method 2: From Method 1, the systolic and diastolic pressures were obtained and the mean arterial pressure (MAP) was calculated (Formula 1). MAP was then used as the constant pressure measurement to determine the heart index and compare it with the 12-lead ECG results at the same time.

Formula 1

(3) Method 3: A constant pressure (60 mmHg) was used to analyze the heart index and to compare it with the simultaneous 12-lead ECG results.

(4) The sensitivity, specificity, PPV, and NPV of AF were analyzed by individual analysis and comprehensive analysis at the end of the trial.

(5) The heart index criteria for judging AF:

(I1 + I2 + I3) >= 5.0 & I1 >= 2.0：AF

(I1 + I2 + I3) <5.0：non- AF

4 ANALYSIS SETS/ POPULATIONS/SUBGROUPS

This unblinded clinical trial was conducted at the Emergency Room of Taipei Medical University Hospital.

Twenty-nine subjects with AF and 33 without AF, aged 25 to 97 years (mean: 63.5 years), were included.

Intervention(s) for clinical trials Subjects who had been exposed to high frequency surgical equipment during testing, those with cardiac pacemakers or implantable defibrillators, and pregnant women were excluded.

4.1 Subjects older than 20 years of age who had been diagnosed with AF using 12-lead ECG, or were healthy (without AF) were included.

4.2 Subjects who had been exposed to high frequency surgical equipment during testing, those with cardiac pacemakers or implantable defibrillators, and pregnant women were excluded.

5 HANDLING OF MISSING VALUES AND OTHER DATA CONVENTIONS

When the test results can not be used as a physician to diagnose the condition of the heart, this data will not be included in the statistical analysis of efficacy evaluation. For unused data, the trial physician must analyze the probable factors that trigger the situation and record it in the case report form.

6 STATISTICAL METHODOLOGY

This clinical trial was conducted in an open study (non-blinded). In the emergency room, when medical staff found patients with AF, they informed the research physicians to study the case. The Heart Spectrum Blood Pressure Monitor was compared with 12-lead ECG (ELI 250) after written informed consent was obtained from the patient. The results were interpreted by the emergency physician. Finally, the sensitivity, specificity, positive predictive value (PPV), and negative predictive value (NPV) were analyzed to evaluate the clinical efficacy.

7 SENSITIVITY ANALYSES

To compare the diagnostic efficacy of the Heart Spectrum Blood Pressure Monitor with a 12-lead ECG in patients with AF. Three measurement methods were used in this study: blood pressure, mean arterial pressure (MAP) which is calculated from individual blood pressure as constant pressure, and a constant pressure 60 mmHg, in order to analyze the heart index and compare it with simultaneous 12-lead ECG.

(1) Method 1: Standard blood pressure measurement was used to determine the heart index and compare with 12-lead ECG synchronously.

(2) Method 2: From Method 1, the systolic and diastolic pressures were obtained and the mean arterial pressure (MAP) was calculated (Formula 1). MAP was then used as the constant pressure measurement to determine the heart index and compare it with the 12-lead ECG results at the same time.

Formula 1

(3) Method 3: A constant pressure (60 mmHg) was used to analyze the heart index and to compare it with the simultaneous 12-lead ECG results.

(4) The sensitivity, specificity, PPV, and NPV of AF were analyzed by individual analysis and comprehensive analysis at the end of the trial.

8 QC PLANS

To ensure that the rights and well-being of the subject are protected, the monitor must confirm that the subject has signed the consent of the subject prior to participating in the trial and that the relevant personnel and test equipment are properly and safely and properly tested.

During the execution of the clinical trial, the monitor will confirm whether the test moderator has complied with the approved test plan and its amendments; report the progress of the admission of the subject; the record of the use of the test equipment; the CRF Registration, original data, archives and all adverse events have been notified as required.

The monitoring frequency is 4 weeks and the test unit shall record the monitoring date in the "monitoring record". During the monitoring process, the relevant investigators must be present and discuss with the monitor the questions found in the process of checking CRF with the original records. In addition, the testing organization must provide a place for the monitor to read the relevant research document or file.

To ensure the quality of the clinical data for each subject, the data manager needs to check whether the case information is consistent or whether there is an omission. In addition, attention should be paid to whether the information is based on the excellent clinical trials of the program books and medical equipment.

9 PROGRAMMING PLANS

|  | Af (+)diagnosed by ECG | non Af (-) diagnosed by ECG |
| --- | --- | --- |
| Test Result(+) by Heart Spectrum Blood Pressure Monitor | A True Positive | B False Positive |
| Test Result(-) by Heart Spectrum Blood Pressure Monitor | C False Negative | D True Negative |
| Sensitivity (Sen.) = A / (A + C)  Specificity (Spe.) = D / (B + D)  PPV = A/ (A + B)  NPV = D/ (C + D) | | |

10 REFERENCES

[1] Feinberg WM, Blackshear JL, Laupacis A, Kronmal R, Hart RG. Prevalence, age distribution, and gender of patients with Af. Analysis and implications. Arch Intern Med. 1995;155(5):469-473.

[2] Wattigney WA, Mensah GA, Croft JB. Increasing trends in hospitalization for Af in the United States, 1985 through 1999: Implications for primary prevention. Circulation. 2003;108:711-716.

[3] Wattigney WA, Mensah GA, Croft JB. Increased Af mortality: United States, 1980–1998. Am J Epidemiol. 2002; 155: 819-826.

[4] Pérula-de-Torres LA, Martínez-Adell MA, González-Blanco M, et al. Collaborative Group DOFA-AP. Opportunistic detection of AF in subjects aged 65 years or older in primary care: a randomised clinical trial of efficacy. DOFA-AP study protocol. BMC Fam Pract. 2012;13:13-106.

[5] Stewart S, Hart CL, Hole DJ, McMurray JJ. A population-based study of the long-term risks associated with Af: 20- year follow-up of the Renfrew/Paisley study. Am J Med. 2002;113:359-364.

[6] Benjamin EJ, Levy D, Vaziri SM, D’Agostino RB, Belanger AJ, Wolf PA. Independent risk factors for Af in a population-based cohort. The Framingham Heart Study. JAMA. 1994;271:840-844.

[7] Wolf PA, Abbott RD, Kannel WB. Af as an independent risk factor for stroke: the Framingham Study. Stroke. 1991; 22(8):983-988.

[8] Benjamin EJ, Wolf PA, D'Agostino RB, Silbershatz H, Kannel WB, Levy D. Impact of Af on the risk of death: the Framingham Heart Study. Circulation. 1998;98(10):946-952.

[9] Conen D, Chae CU, Glynn RJ, et al. Risk of death and cardiovascular events in initially healthy women with new-onset Af. JAMA. May 25, 2011;305(20):2080-2087.

[10] Wang TJ, Larson MG, Levy D, et al. Temporal relations of Af and congestive heart failure and their joint influence on mortality: the Framingham Heart Study. Circulation. 2003;107(23):2920-2925.

[11] Conen D, Osswald S, Albert CM. Epidemiology of Af. Swiss Med Wkly. 2009;139(25–26):346-352.

[12] Warren JM, Daniel ES, Gregory YHL, et al. Af: Anticoagulant therapy to prevent embolization. ©UpToDate. August, 2016.

[13] Fung E, Järvelin MR, Doshi RN, et al. Electrocardiographic patch devices and contemporary wireless cardiac monitoring. Front Physiol. 2015;6:149.

[14] Camm AJ, Lip GY, De Caterina R, et al; ESC Committee for Practice Guidelines (CPG). 2012 focused update of the ESC Guidelines for the management of Af: an update of the 2010 ESC Guidelines for the management of Af. Developed with the special contribution of the European Heart Rhythm Association. Eur Heart J. 2012;33(21):2719-2747.

[15] Rosero SZ, Kutyifa V, Olshansky B, Zareba W. Ambulatory ECG monitoring in Af management. Prog Cardiovasc Dis. 2013;56(2):143-152.

[16] Barrett PM, Komatireddy R, Haaser S, et al. Comparison of 24-hour Holter monitoring with 14-day novel adhesive patch electrocardiographic monitoring. Am J Med. 2014;127(1):95.e11-17.

[17] Joseph W. Home Monitoring for Af Using a Microlife Blood Pressure Monitor (TRIPPS.) ClinicalTrials.gov Identifier: NCT00861354. March 10, 2009.

[18] Riyaz AK. Six Lead Identification of Af (SL-AF.) ClinicalTrials.gov Identifier: NCT02124629. March 24, 2015.

[19] Ajijola OA, Boyle NG, Shivkumar K. Detecting and monitoring arrhythmia recurrence following catheter ablation of Af. Front Physiol. 2015; 6:90.

[20] Barrett PM, Komatireddy R, Haaser S, et al. Comparison of 24-hour Holter monitoring with 14-day novel adhesive patch electrocardiographic monitoring. Am J Med. 2014;127(1):95.e11-17.

[21] Higgins P, MacFarlane PW, Dawson J, McInnes GT, Langhorne P, Lees KR. Noninvasive cardiac event monitoring to detect Af after ischemic stroke: a randomized, controlled trial. Stroke. 2013;44(9):2525- 2531.

[22] Vaes B, Stalpaert S, Tavernier K, et al. The diagnostic accuracy of the MyDiagnostick to detect Af in primary care. BMC Fam Pract. 2014;15:113.

[23] Tieleman RG, Plantinga Y, Rinkes D, et al. Validation and clinical use of a novel diagnostic device for screening of Af. Europace. 2014;16(9):1291-1295.
